# Targeted Feature Recognition Using Mechanical Spatial Filtering with a Low-Cost Compliant Strain Sensor

**DOI:** 10.1038/s41598-017-05341-w

**Published:** 2017-07-11

**Authors:** Eli M. Barnett, Julian J. Lofton, Miao Yu, Hugh A. Bruck, Elisabeth Smela

**Affiliations:** 1 Department of Mathematics, University of Maryland, College Park, MD 20742 USA; 2 Department of Mechanical Engineering, University of Maryland, College Park, MD 20742 USA; 3 Institute for Systems Research, University of Maryland, College Park, MD 20742 USA

## Abstract

A tactile sensing architecture is presented for detection of surface features that have a particular target size, and the concept is demonstrated with a braille pattern. The approach is akin to an inverse of mechanical profilometry. The sensing structure is constructed by suspending a stretchable strain-sensing membrane over a cavity. The structure is moved over the surface, and a signal is generated through mechanical spatial filtering if a feature is small enough to penetrate into the cavity. This simple design is tailorable and can be realized by standard machining or 3D printing. Images of target features can be produced with even a low-cost compliant sensor. In this work a disposable elastomeric piezoresistive strain sensor was used over a cylindrical “finger” part with a groove having a width corresponding to the braille dot size. A model was developed to help understand the working principle and guide finger design, revealing amplification when the cavity matches the feature size. The new sensing concept has the advantages of being easily reconfigured for a variety of sensing problems and retrofitted to a wide range of robotic hands, as well as compatibility with many compliant sensor types.

## Introduction

Tactile sensing for robots has been the subject of excellent and comprehensive reviews^1–8^. Considering only devices that can be placed directly on the gripping surface of a robot finger^1^, tactile sensors can be broadly divided into area sensors, used for exploring shape or relatively large features, and line or point sensors that scan over a surface, used for exploring texture and other fine features^3^. Area sensors span a wide range of complexity and sophistication, from piezoresistive matrix grids^9, 10^, to microfabricated silicon pixel arrays^11^, flexible micromachined polymer “skins”^12–14^, and complete sensing finger systems^15^. A few studies have reported images produced when the sensors were brought into contact with an object^16–20^. Despite the many well-performing tactile sensors that have been developed, their use on robot hands has not been widely adopted^7^, partially due to cost and difficulty of integration. For these reasons, the detection of fine detail with robot hands remains limited^3^.

For human fingers to explore texture, they are moved over a surface. Dynamic tactile sensing is defined as sensing during motion^1, 21^. Scanning requires only a single sensor or line of sensors to create an image, but it also requires accurate three-degrees-of-freedom positioning. Two of the design principles for dynamic tactile sensing are (1) mechanical isolation, so that signals come only from a location of interest, and (2) event correlation, so that signals are generated only in response to a particular type of event^22^. Both are applied in the present work.

Compliance confers fingers with advantages in grasping^2, 23^, so the use of soft materials in tactile sensors has become a focus of research^4, 6, 8, 24^. Compliant sensors are often strain sensors, indicating force indirectly. They are typically viscoelastic and thus experience time-dependent behavior, including hysteresis. Nevertheless, they can function adequately because for measuring contact *displacements*, sensor mechanical properties are not as critical. The low force required to displace a soft sensor is advantageous for probing pliable surfaces. Texturing compliant surfaces has been found to enhance sensitivity^25, 26^ and resolution^2^ by increasing local strain. This work takes texturing to a new level.

**Figure 1:**
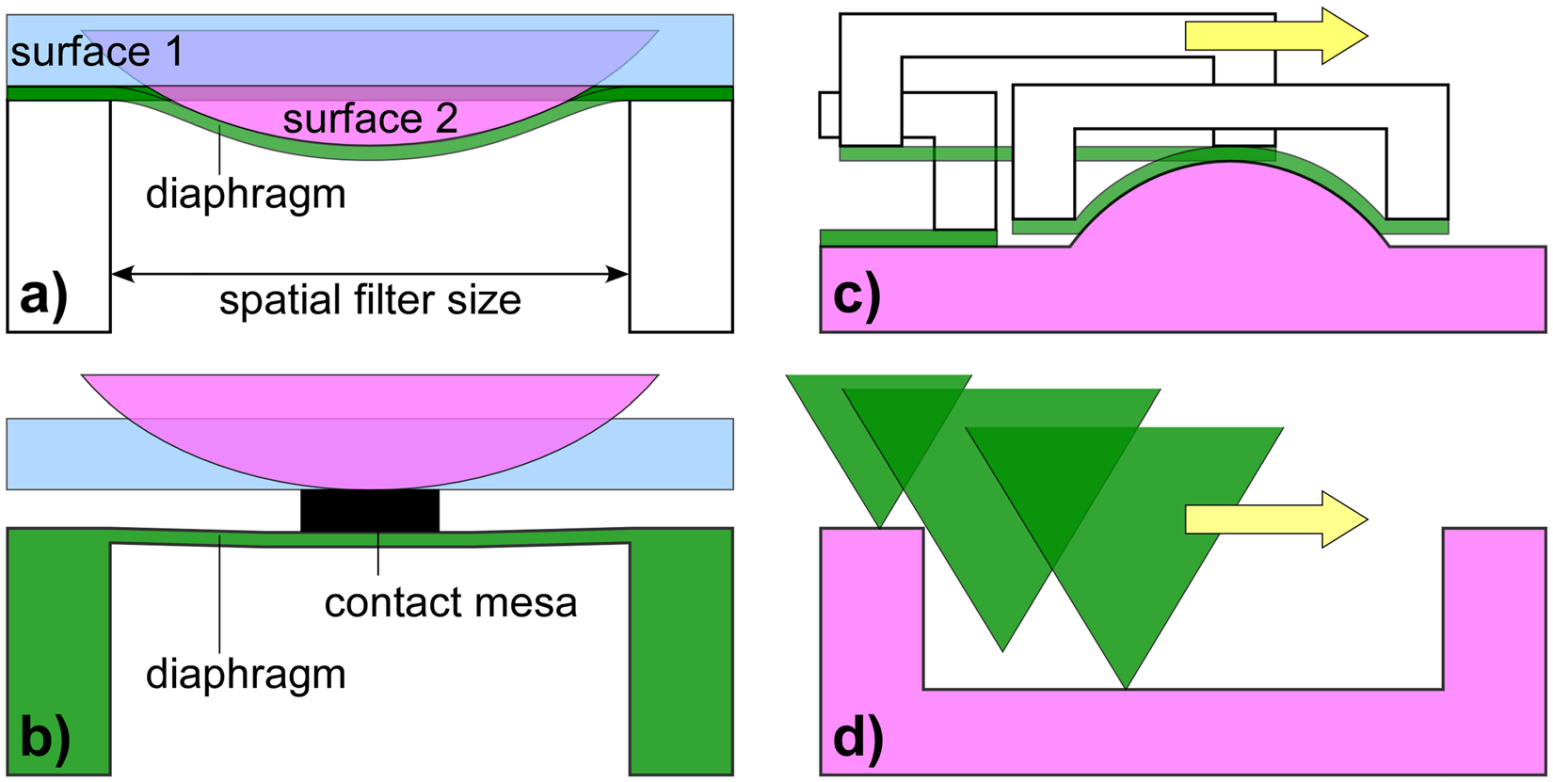
(**a**) Our architecture implementing mechanical spatial filtering. Contact with a flat surface (surface 1) does not deflect the diaphragm, but a large signal is generated when a surface feature of matching size (surface 2) is encountered. (**b**) A standard diaphragm-type sensor. A signal is generated by pressure from a flat surface as well as one with a curved feature. (**c**) Our concept resembles a “reversed” profilometer, in which the indenting object is imaged by a deflecting surface. (**d**) Standard mechanical profilometry, in which a tip scans over a surface to obtain its shape.

In prior work on compliant piezoresistive sensors, we showed that localizing strain results in an increased sensor output^27^. This paper explores the use of the localization principle to realize a new displacement-based sensing architecture. The focus of this paper is the design of the sensing structure (Fig. 1a,c) and the study of its interaction with surface features, both experimentally and theoretically.

**Figure 2:**
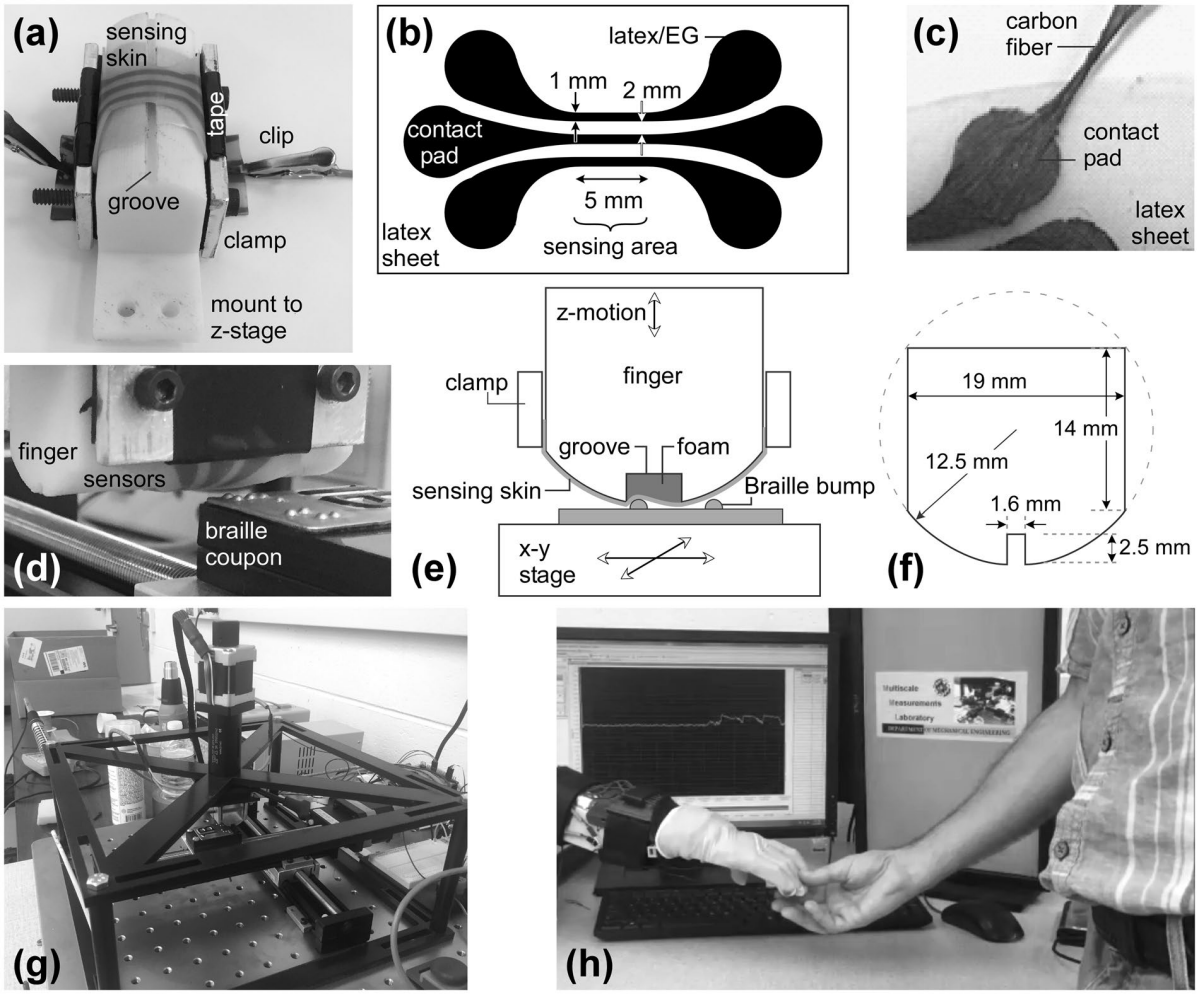
(**a**) A 3D printed finger with the sensing membrane clamped over it. (**b**) Schematic showing three sensing strips. (**c**) A carbon fiber bundle electrically and mechanically connected to the sensor. (**d**) A finger positioned over the braille coupon. (**e**) Schematic showing the components of the machined finger with a foam-filled groove of 5 mm width. (**f**) Dimensions of the 3D printed finger shown in (**a**). (**g**) Overview of the test rig showing the *x*-*y* and *z* positioners, crossbar support, and, in the center, the finger and braille coupon. (**h**) Hand of a robot arm wearing a sensorized glove, with the finger detecting contact with a human.

The sensing hardware consists of a stretchable sensor mounted over a finger-shaped part with a cavity (Fig. 2a). The finger is moved over a surface, and when a feature fits into the cavity the sensor is deflected, resulting in a signal. The cavity acts as a high-pass “spatial filter” in which pressure from a flat surface (a DC signal) and features larger than the groove (low frequency) are screened out. This is useful for a subset of imaging tasks in which features of a particular size are sought.

In this work the sensing material was a composite of latex filled with nano-scale carbon, in which electrical conductivity is governed by percolation. Such sensors have been extensively characterized in our prior work^27–32^ and by others^33–44^, and therefore are not the topic of this work. The piezoresistive composite has a linear response to at least 25% strain and a constant gauge factor on the order of 14^27^. The composite was applied as a thin film^27, 29, 30^ onto a latex rubber membrane.

The new structure was demonstrated through experiments with a standard braille pattern. (Note that the structure is not proposed as a braille-reading device: while tactile sensors have been suggested for this purpose^45, 46^, optical imaging is an alternative approach that may be faster^3^). This task was faultlessly performed: the braille images were remarkably robust to variations in the sensing layer, contact depth, and manner of scanning. Although a single rudimentary piezoresistive sensor was used here, the finger structure could employ other sensing modalities, such as capacitive or piezoelectric, or multi-pixel stretchable sensors. It could also be designed to have multiple spatial filters to match different feature sizes on a geometrically complex surface. This work thus has the potential to be transformative for robotic applications. The sensing concept is simple, low cost, retrofittable to a range of robotic hands, and compatible with a variety of sensor types.

Compared with a standard diaphragm-type strain gauge sensor suspended over a cavity (Fig. 1b), used for measuring pressure, force, or hardness^5, 12, 16, 47, 48^, our tactile imaging system offers two unique features. First, an elastomeric sensor allows a large displacement (Fig. 1a), producing a correspondingly large signal. Second, the diaphragm sits flush with the surface, rather than including a contact mesa, so only protrusions from the scanned surface lead to a signal. The sensor is conceptually similar to mechanical profilometry or atomic force microscopy in which a sharp probe tip is scanned over a surface to produce an image of the surface topography. In profilometry the scan profile is distorted by the probe shape due to steric hindrance (Fig. 1d)^49^. In our sensor (Fig. 1c) the surface feature switches roles with the probe, as discussed below.

## Results

### Device fabrication and test rig

Different types of fingers were fabricated and tested (Fig. 2e,f), all of which were covered with a sensing membrane with three parallel sensing strips (Fig. 2b). The sensing strips were made by spray coating an aqueous solution of latex and exfoliated graphite (EG) through a stencil onto a latex rubber membrane, as summarized in Methods and described in detail in refs 27–30, 32. Carbon fiber leads were connected to the sensor (Fig. 2c) by employing the same latex/EG solution as an adhesive (Fig. 2c). The low-cost sensing membrane can be readily replaced, making it disposable. One finger was machined from acrylic to have a cylindrically-fronted surface and a 5 mm wide groove, which was filled with a finely-textured open-cell foam (Fig. 2e). Another was a fused deposition modeling (FDM) printed acrylonitrile butadiene styrene (ABS) finger (Fig. 2a,f). (A third finger and the results from it are shown in Supplementary Figures S21 and S22).

**Figure 3:**
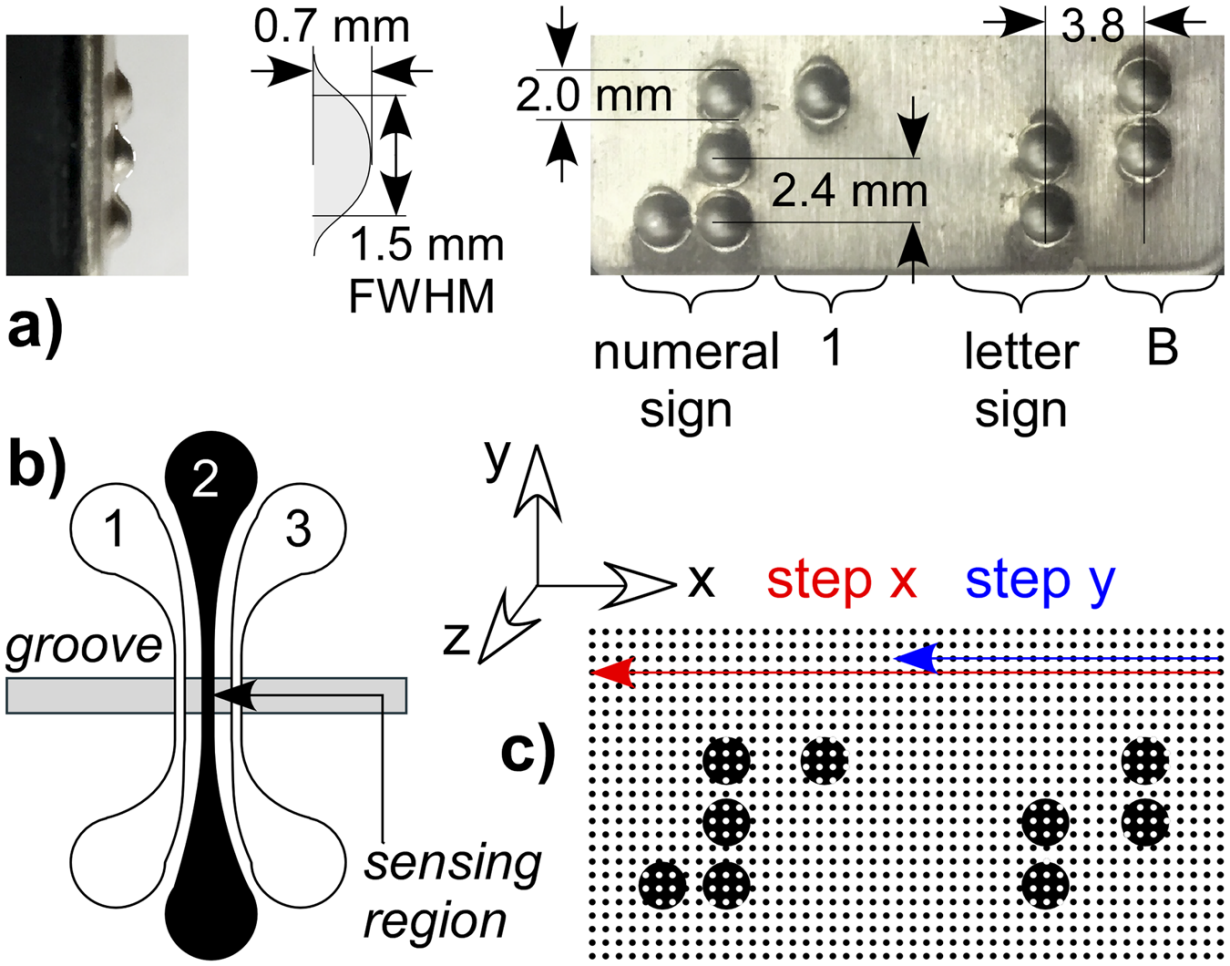
(**a**) Edge-on close-up of the stamped metal braille coupon, dimensions of the braille dots, and overhead close-up of the braille surface. (**b**) Schematic of the sensing strips over the groove; the latex membrane is not shown. Images were typically obtained using just one of the three sensors, labeled 1–3. (**c**) For the discrete scans, the sensor was stepped from right to left in the *x*-direction over the target by 0.5 mm increments, moved back to the right edge and up by 0.5 mm in the *y*-direction, and stepping in *x* begun again.

The braille target consisted of a pattern of bumps 2 mm in diameter (Figs 2d and 3a). The target and the finger were mounted on a motorized test bed (Fig. 2e,g) consisting of an *x-y* positioning table and a vertical *z*-axis actuator; this microprocessor-controlled rig served as a robot arm simulator. In principle, the sensing finger could be mounted to a robot hand such as the one shown in Fig. 2h. Two types of scans were performed to acquire a tactile image of the braille: “discrete” scans (Fig. 3c) in which the braille plate was probed by the sensor at defined points along a grid and “continuous” scans in which the sensor was held at a fixed height and swiped along the braille plate in a set of parallel lines.

### Discrete scans

**Figure 4:**
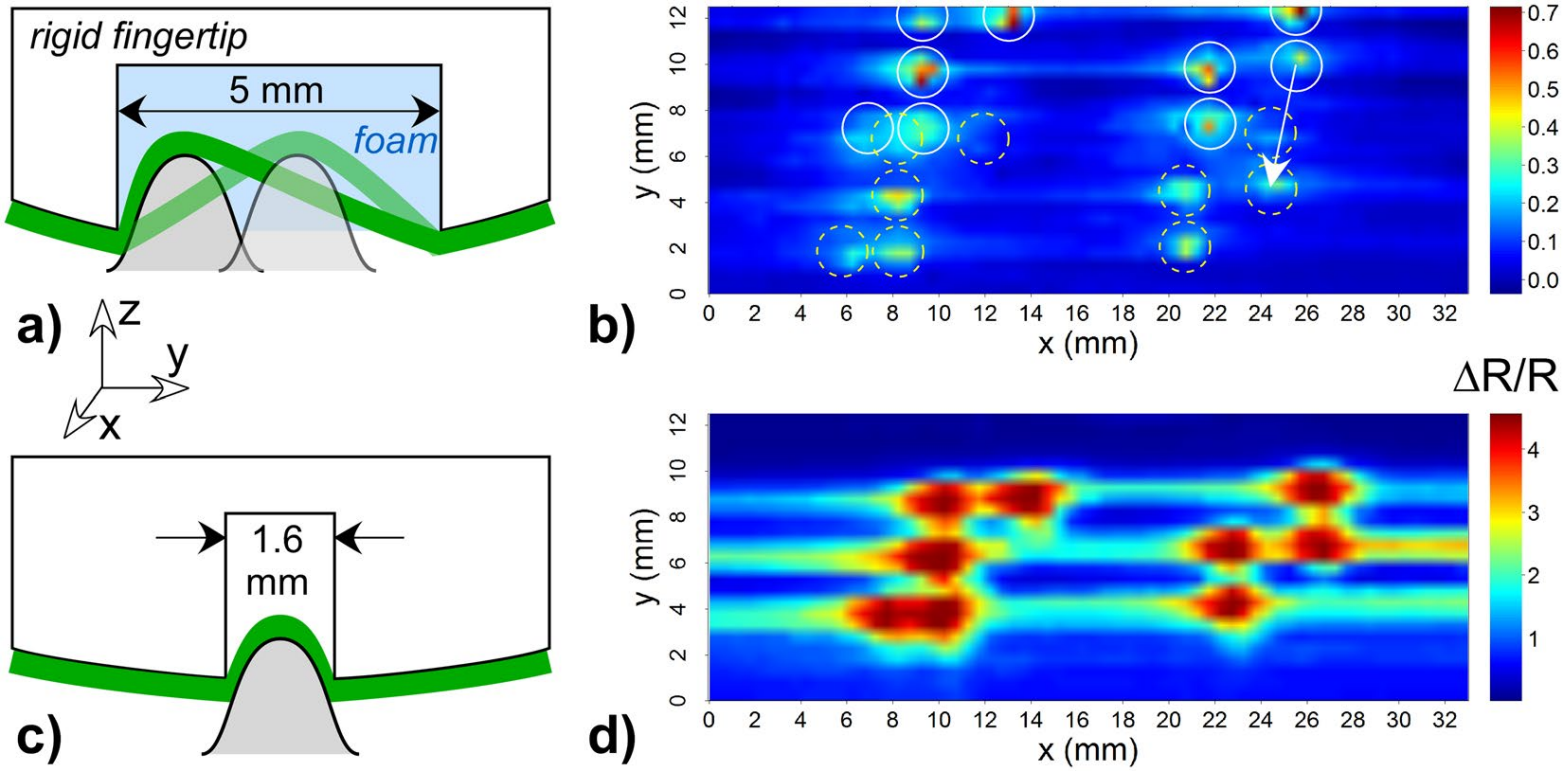
(**a**) Schematic representation of the first finger, with a foam-filled groove, and the interaction of a braille dot with the sensing membrane, represented as a green line. The vertical scale is exaggerated for clarity. (**b**) The corresponding discrete scan image. The peak ΔR/R values for each touch are indicated by the color, with maximum values in red and the baseline in blue. (Note: the color scales in the images vary.) The *x*-direction in the image is along the groove. The braille pattern is overlaid (circles with solid lines), as is ghosting (dashed lines). (**c**) A finger with a narrower, air-filled groove. (**d**) The corresponding discrete scan image.

The aim of the discrete scans was to determine whether the system had sufficient sensitivity to visualize the braille pattern. In prior work we showed that a foam padding layer under a sensing membrane results in localized strain, amplifying the sensor response, while compression of the membrane over a solid support generates no significant signal^27^. Thus, scans were first performed with a machined finger having a foam-filled groove beneath the sensing membrane, the groove oriented parallel to the *x*-direction. The scans involved lowering the finger vertically (along the *z*-axis) to a fixed distance from the surface baseline to take a measurement, raising the finger, and then incrementing the *x,y* position, systematically covering the area of the braille pattern (Fig. 3c). Replicate runs were performed on two sensing membranes. The best image is shown in Fig. 4b. (Other images are shown in Supplementary Figure S20).

The foam-filled finger did have the required sensitivity and resolution: peaks were obtained at the exact positions corresponding to the braille dots, as shown by the white circles overlaid on the image. The signal was large: peak heights were up to ΔR/R = 0.7. Although there was some smearing to the right of the dots due to viscoelastic memory effects (Supplementary Figure S14), the signal returned nearly to the baseline between dots. The peaks were of varying amplitude, with the left-most peak being quite faint; as discussed below, this reflected actual variations in the dot heights. However, the image appeared in duplicate, with a “ghost” image systematically offset by 5.3 mm in the *y*-direction and 1.2 mm in *x*, as indicated by the arrow to the dashed circles. The reason for the double image in the *y*-direction is illustrated in the accompanying schematic (Fig. 4a) and discussed in the *Modeling contact* section: the strongest signal was obtained not from the center of the groove, but from both edges. (The small separation distance in the x-direction is likely due to the sensing strip not being exactly perpendicular to the groove and the sampling points not being aligned with the dot centers). From these results, it was postulated that the groove needed to be made narrower, to correspond to the dot width.

Localization can also be achieved with a narrow air gap under a sensing membrane^27^. A simpler finger (Fig. 4c) was thus fabricated, without foam, using 3D printing. The groove was 1.6 mm wide, a value just greater than the 1.5 mm full width at half maximum (FWHM) of the dots (Fig. 3a).

Discrete images were obtained as before (Fig. 4d). The braille pattern is again clearly seen, with good peak heights for all the dots and, importantly, no ghosting. The signal amplitude was significantly larger from this configuration due to nonlinear amplification, as discussed below. The round dots appeared wider in *x* (along the groove) than in *y* (perpendicular to it) due to the asymmetry of the groove-shaped cavity.

### Modeling contact

In prior work we showed that under biaxial strain, the signal from our sensor scaled with the linear sum of the strains in orthogonal directions^27^. The sensors used here had the shape of a thin strip (1 mm wide) suspended over a groove (1.6 mm wide) with vertical sidewalls (Fig. 2a). The membrane on which the sensors were patterned had a width of 1–2 cm. For a maximum displacement of 0.6 mm into a groove of 1.6 mm, the strain along the sensor is 25% $((2\sqrt{{0.6}^{2}+{0.8}^{2}}\,-\,1.6)/1.6)$ while the strain perpendicular to it is less than 1% $((2\sqrt{{0.6}^{2}+{5}^{2}}\,-\,10)/10)$. Under these conditions, the problem can be reasonably considered as 2D.

The membrane is soft compared to the the feature and the groove, providing negligible mechanical resistance to motion; therefore, to first order only displacement needs to be considered. The groove is treated as a rectangular notch that is able to move up and down in the *z*-direction to maintain contact with the feature surface, but it is not able to rotate. (In the physical system, this motion in *z* was enabled by flex in the test rig frame, Fig. 2g).

**Figure 5:**
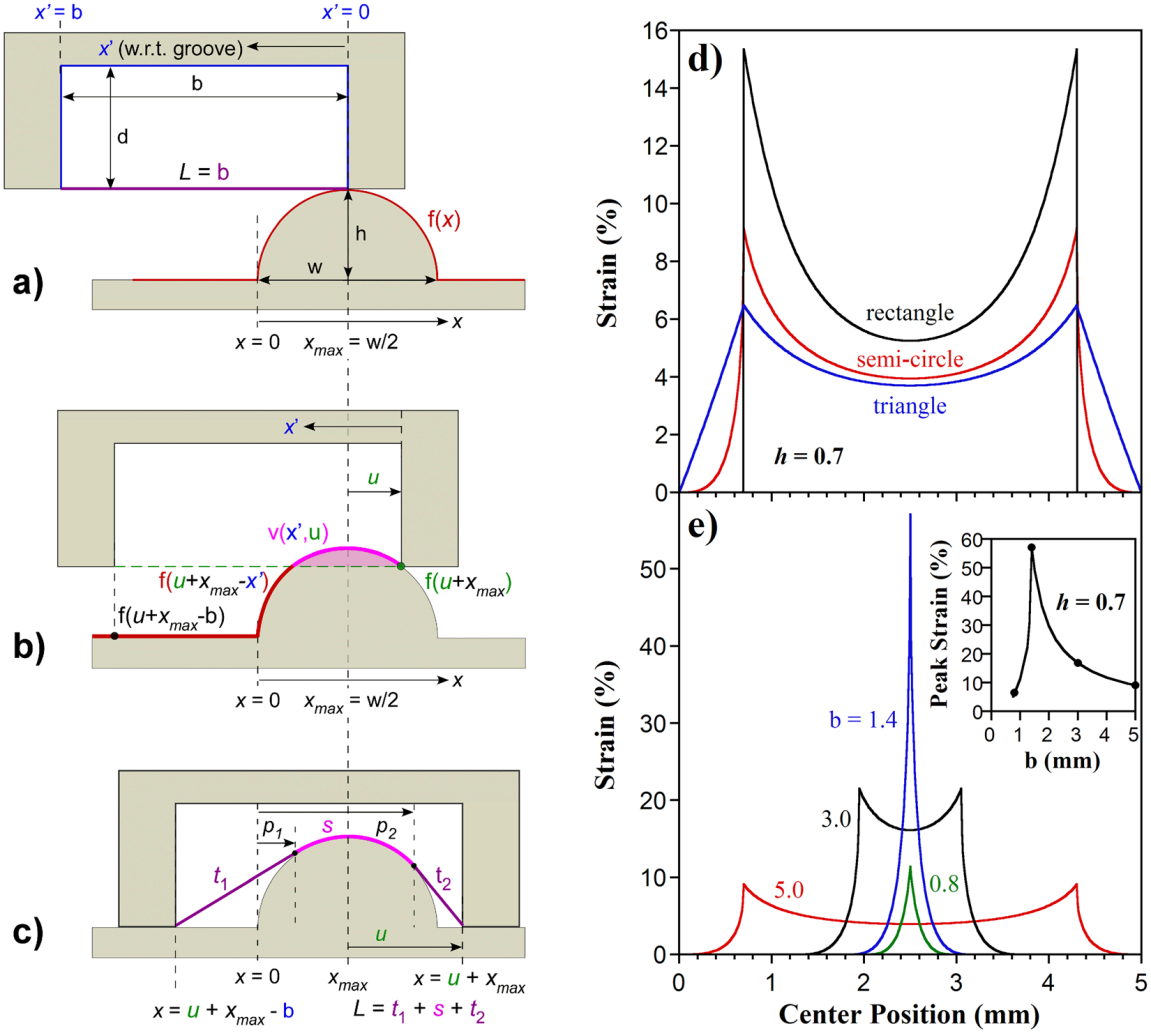
(**a**) Illustration of the mathematical model for a semicircular feature shorter and narrower than a notch. The notch begins at the highest point on the feature and moves an amount 0 < *u* < b in the *x*-direction. Coordinates with respect to the groove are given in the moving coordinate system *x*′. (**b**) As the notch moves, the feature penetrates into it as described by *v*(*x*′,*u*). (**c**) The stretched membrane has a region in contact with the feature and may have one or two regions that are suspended. (**d**) Model predictions of sensor strain as a function of feature position for three different feature shapes, all with width *w* = 1.4 mm and height *h* = 0.7 mm, within a rectangular notch of breadth *b* = 5 mm and depth *d* > 0.7 mm. (**e**) Predictions for a semi-circular feature of *w* = 1.4 mm and *h* = 0.7 mm in 4 notch breadths: 5, 3, 1.4, and 0.8 mm. (inset) Peak strain as a function of notch breadth, showing nonlinear amplification. The points indicate the four cases shown in (**e**).

To model the strain in the sensor, we first determine the contact between the notch and a feature described by a general function f(*x*), and then determine the shape of a membrane connected to the convex inner corners of the notch. The feature has width *w* and height *h* and sits in a fixed reference frame *x* (Fig. 5a). The notch has breadth *b* and depth *d* and starts with its inner right hand side (r.h.s.) at position *x*_max_, the location of the greatest value of f(*x*). Let *x*′ be a moving reference system defined with its origin at the inner right-hand side of the notch. The notch is then moved horizontally by an amount *u* along *x*, shifting downward in *z* at the same time. The origin of *x* and *x*′ are coincident when *u* = 0. When *u* = b, the inner left hand side (l.h.s.) of the notch arrives at *x*_max_.

We start with the case that *d* > *h* and *b* > *w* (Case 1), which corresponds to our physical system. There is no membrane strain until *u* > 0. The vertical displacement field *v* describes the depth of the feature within the notch and is a function of *u* as well as position *x*′ within the notch. Since contact with the feature is maintained as the notch moves rightward and downward, *v* is given by the height of the position above the contact point (Fig. 5b):

1 $$v(x\text{'},u)=f(u+{x}_{max}-x\text{'})-f(u+{x}_{max})\,{\rm{f}}{\rm{o}}{\rm{r}}\,v > 0.$$

The elastic membrane maintains contact with the feature unless the surface of the feature goes below a line *t* connecting a point on the surface *p* with the inside corner of the notch. This occurs when the surface tangent at *p* is equal to the slope of that line (Fig. 5c). Thus, to the right of *x*_max_, where $0 < x\text{'} < u$, there is a “take-off” point *p*_2_ if

2 $$\frac{df(u+{x}_{{\rm{\max }}}-x\text{'})}{dx}\le -\frac{v(x\text{'},u)}{x\text{'}},$$

at the point where these two terms are equal. Likewise, for $u < x\text{'} < b$, there will be a point *p*_1_ if

3 $$\frac{df(u+{x}_{{\rm{\max }}}-x\text{'})}{dx}\ge \frac{v(x\text{'},u)}{b-x\text{'}}$$

at the position where the equality holds true. At *p*_2_, the displacement is

4 $$v({p}_{2},u)=f({p}_{2})-f(u+{x}_{{\rm{\max }}}),$$

and to the right of *p*_2_ it increases linearly with *x*′:

5 $$v(x\text{'},u)=\frac{x\text{'}}{(u+{x}_{{\rm{\max }}})-{p}_{2}}[f({p}_{2})-f(u+{x}_{{\rm{\max }}})].$$

The inner two convex notch corners are at the same height, $f(u+{x}_{{\rm{\max }}}-b)=f(u+{x}_{{\rm{\max }}})$. Thus, to the left of *p*_1_ the displacement decreases linearly with *x*′ as described by:

6 $$v(x\text{'},u)=\frac{b-x\text{'}}{{p}_{1}-(u+{x}_{{\rm{\max }}}-b)}[f({p}_{1})-f(u+{x}_{{\rm{\max }}})]$$

Between *p*_1_ and *p*_2_, the displacement is given by equation (1). The total deformed length of the membrane is found by integration and then adding the length of the sensor that extends beyond the notch to the electrical connections, *L*_ex_:

7 $${L}_{d}(u)={\int }_{0}^{b}\sqrt{1+{(\frac{dv(x\text{'},u)}{dx\text{'}})}^{2}}dx\text{'}+{L}_{ex}.$$

The average engineering strain throughout the sensor is then given by *ε*(*u*) = (*L*_d_(*u*) − *L*_0_)/*L*_0_, where *L*_0_ = *b* + *L*_ex_ is the original sensor length. The change in output signal is found using the gauge factor if the output is linear with strain.

These equations hold when *u* + *x*_max_ < *b*. When *u* + *x*_max_ = *b*, the left inner corner of the notch touches the feature, and the notch begins to rise again. Then the r.h.s. contact point f(*u* + *x*_max_) needs to be changed to the l.h.s. contact point f(*u* + *x*_max_ − *b*) if the feature is asymmetric. This will be valid until *u* = *b*, at which point the sensor strain is 0 again.

There are three other cases to consider. (Case 2) When *b* < *w* and *d* > *h*, the feature is wider than the notch but still shorter. The solution is the same as case 1 except that the condition for f(*u* + *x*_max_) changing to f(*u* + *x*_max_ − *b*) occurs when f(*u* + *x*_max_) < f(*u* + *x*_max_ − *b*). (Case 3) When *b* > *w* and *d* < *h*, the feature is narrower than the notch but taller. The solution is again the same as case 1 except that for *h* − f(*u* + *x*_max_) > *d*, f(*u* + *x*_max_) becomes *h* − *d*. (Case 4) When *b* < *w* and *d* < *h*, the feature is wider and taller than the notch. The solution is the same as case 2 except that, as in case 3, when *h* − f(*u* + *x*_max_) > *d*, f(*u* + *x*_max_) becomes *h* − *d*.

Examples showing the above model in detail for triangle and semi-circle features are provided in the Supplementary Information (Supplementary Figures S1, S4–S7). In some cases it is simpler to instead construct a geometric model (Supplementary Figures S2, S3, S8, S11), which gives identical results (a comparison is shown in Supplementary Figure S9). The results of modeling membrane strain due to features with different shapes and due to notches of different widths are shown in Fig. 5d,e.

Comparing the strain vs position profiles for semi-circular, triangular, and rectangular cross sections of the same height and width (Fig. 5d), several observations can be made. All of the curves peak at the same distance from the notch edges, 0.7 mm in this example, the point when the feature first reaches, or last maintains, its maximum penetration depth. The strain decreases again as the feature approaches the center of the notch. The rectangle results in the most displacement, followed by the semi-circle and then the triangle, consistent with their areas within the notch.

As established in profilometry^49^, the shape of the feature can be inferred directly from the signal profile at the step edges. Between *u* = 0 and *u* at the peak, strain from a rectangle increases stepwise from 0 to the peak; from a semi-circle it follows a rounded curve; and from a triangle it increases linearly.

Figure 5e shows, for a semi-circular feature of constant size, that when the width of the groove is decreased, the strain increases and the peak separation decreases. When the feature width matches the groove, only a single peak is observed and the strain is maximized. Mechanical filtering (Supplementary Figure S10) thus maximizes the output signal. Further decreasing the notch width, a single peak is maintained, but its amplitude is smaller because less of the feature can fit into the notch. The inset shows the peak strain as a function of notch width, illustrating a nonlinear amplification achieved by spatial filtering, which makes the system more responsive to features of the target size. For a target feature that does not fully penetrate into the notch, the nonlinear amplification also occurs with increasing depth of contact (Supplementary Figures S11, S12).

These models provide a predictive capability and correlate with the observed experimental phenomena. There may be other contributions to the signal, for example from squeezing the membrane at the corners, but these would be smaller. The strain profiles for features narrower than the groove account for the ghosting phenomenon seen in Fig. 4a: the strain at the groove edges is larger, producing two peaks. As the notch decreases in width, the result is more closely spaced peaks, reaching the limit of a single peak when the object size matches the groove. The distance to the ghost signal can therefore in principle be used to determine the size of an object smaller than the groove.

### Continuous scans

Discrete scans were performed by tapping the surface, which is unlike the way people explore surface texture by running a finger along it. The goal of continuous scanning was therefore to determine whether the pattern could be visualized with continuous motion in a set of parallel lines. The same response was not necessarily expected from the viscoelastic membrane, since a transverse force could be imparted by riding over the bumps, which introduces shear. Nevertheless, the device also worked when used this way.

For the continuous scans, the area was mapped line by line (25 total), each line consisting of a forward and reverse swipe over the braille coupon at a fixed height above it (not resting on it), to avoid the possibility of tugging on the membrane. Images were created using the printed finger and a second, nominally identical, braille target. The scanning motion of the finger was in the *x*-direction, and the groove was oriented parallel to the *y*-direction. Flex in the frame on which the *z*-transducer was mounted (Fig. 2g) allowed the finger to ride over the tips of the dots.

**Figure 6:**
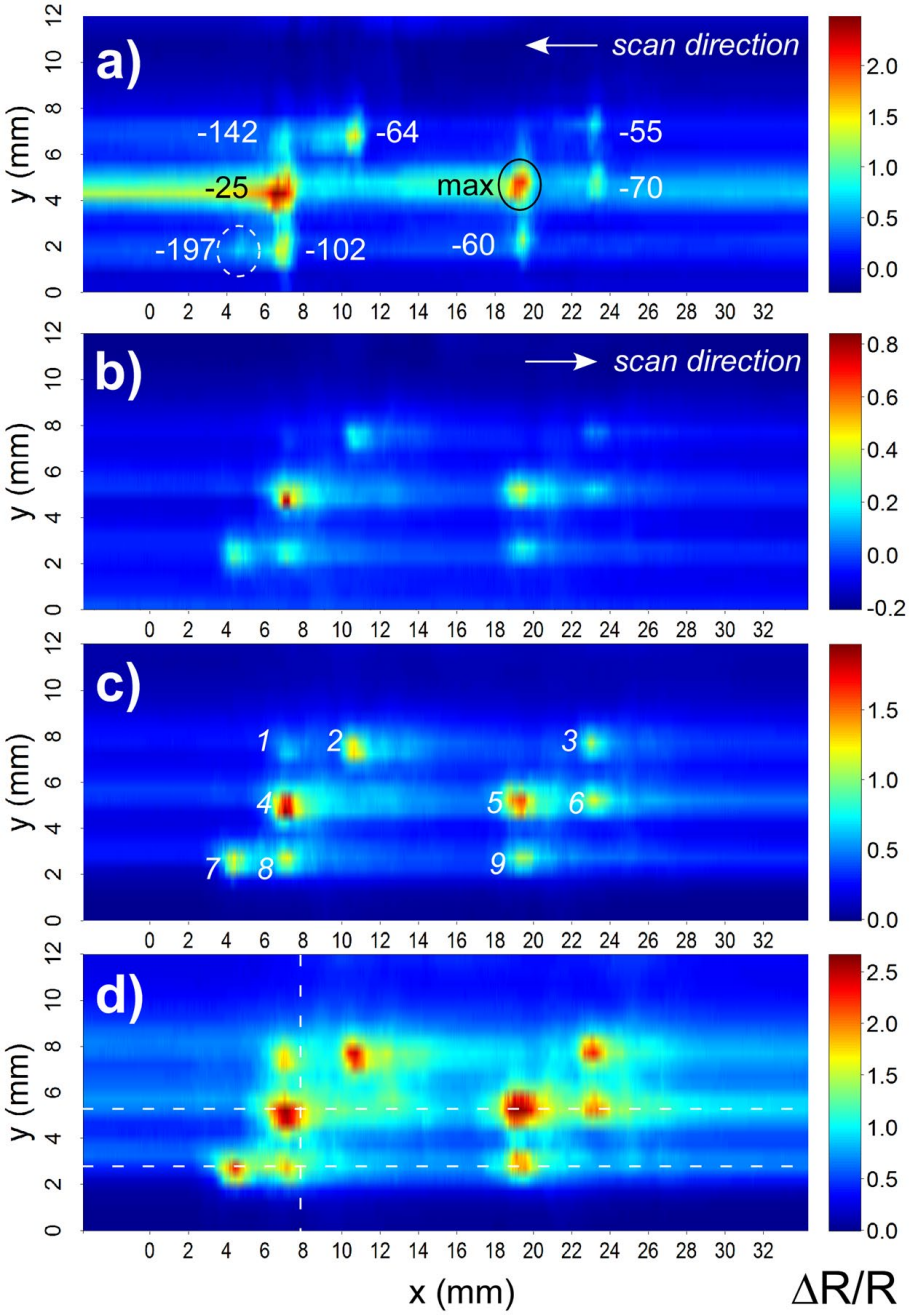
Images obtained from continuous scanning, using the finger illustrated in Fig. 4c. (**a**) The peak heights (μm) of the braille dots on this plate relative to the dot with the largest height are indicated. Scanning was conducted with a shallow contact. The image revealed height differences among the braille dots. (**b**) A scan performed with another sensor. (**c**) Increasing the contact depth used in (**b**) by 100 μm, and (**d**) increasing the depth by another 100 μm. Dot numbers and line scan positions used in Fig. 7 are indicated. Different color scales were used to permit visualization.

The braille pattern is clearly visible in Fig. 6a. The peaks were smaller than in Fig. 4 due to the more shallow depth of contact (the exact distance above the surface is unknown). The streaking was also lower, for the same reason. We noticed that some of the dots were significantly brighter than others, while one of them was nearly invisible. Dot heights were therefore measured. The tallest dot, marked “max”, had a height from the flat bottom surface of 725 ± 10 μm. The height differences in μm for the other dots are shown in the figure. The two brightest dots were the tallest, with nearly the same height. The bottom-left-most dot, almost too faint to see, was the shortest. The sensor thus showed a more accurate representation of the surface than we had expected. (A tilt of the coupon relative to the rig would explain the systematic lowering of signal intensity on the right hand side, since the height was measured with respect to a point on the surface between the two tallest dots).

### Effect of scan depth

Scans were run at three depths of contact, increasing from the first scan by 100 μm (Fig. 6b–d). Ideally, the finger would rest on the surface and ride over it, but further increasing the contact depth introduced too much friction to allow sliding.

As expected, with increasing depth of contact, and thus sensor deflection, the dots became brighter and wider. The streaking also became more significant, as seen previously. Visually observing the sensor during the scan, it was evident that for the greatest depth of contact the dots began to tug at the membrane, and that stick-slip was occurring. Nevertheless, the patterns were evident in all three images (repeatability 100%), suggesting that more precise height control is unnecessary.

**Figure 7:**
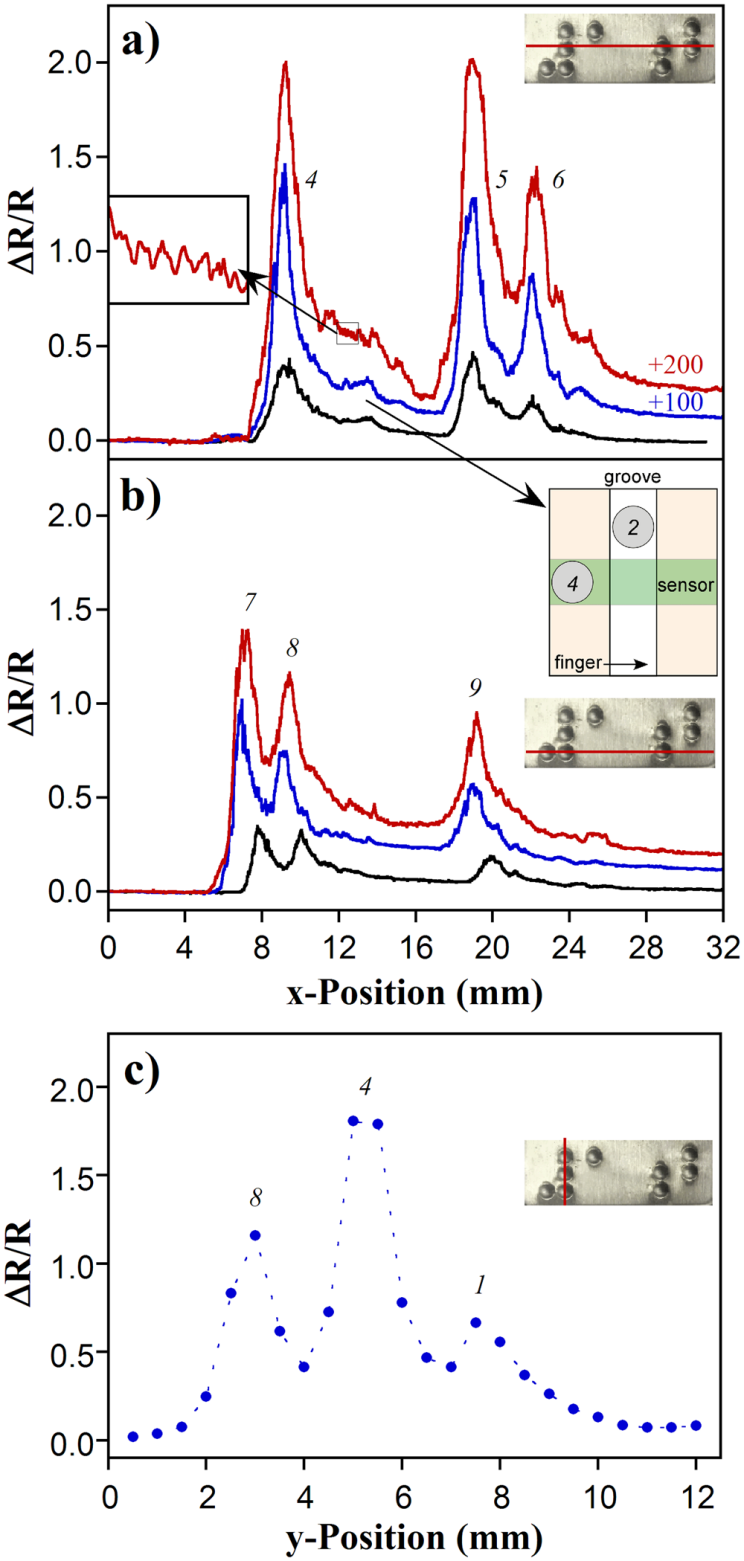
Horizontal lines from the data in Fig. 6b–d, recorded at depths of contact offset by 100 μm, taken at (**a**) *y* = 5.25 mm (center row of dots) and (**b**) *y* = 2.75 mm (bottom row of dots). (**c**) Vertical line from the data in Fig. 6d at *x* = 8 mm (2^nd^ column of dots). (Positions are shown by red lines over the braille coupon here and as dashed lines in Fig. 6d. Dot numbers are shown in Fig. 6c).

The images in Fig. 6 were produced from 25 line scans spaced 0.5 mm apart. The *x*-direction lines through the center of the braille pattern, across the tallest two dots, are shown in Fig. 7a. This figure allows the relative amplitudes of the signals to be compared, since they are on the same vertical scale, showing how much the signal increased with depth of contact. Importantly, the three peaks due to the braille dots are visible in all cases, demonstrating the robustness of the system to differences in depth of contact. As is clear from the inset showing a close-up view, what appears to be increasing noise with greater contact depth is actually instead an increase in stick-slip events, as evidenced by the regular size and frequency of the ripples. Based on the curved shapes of the peaks (Fig. 7a,b), it can be seen that the braille dots are rounded (consistent with the model prediction in Fig. 5d,e).

**Figure 8:**
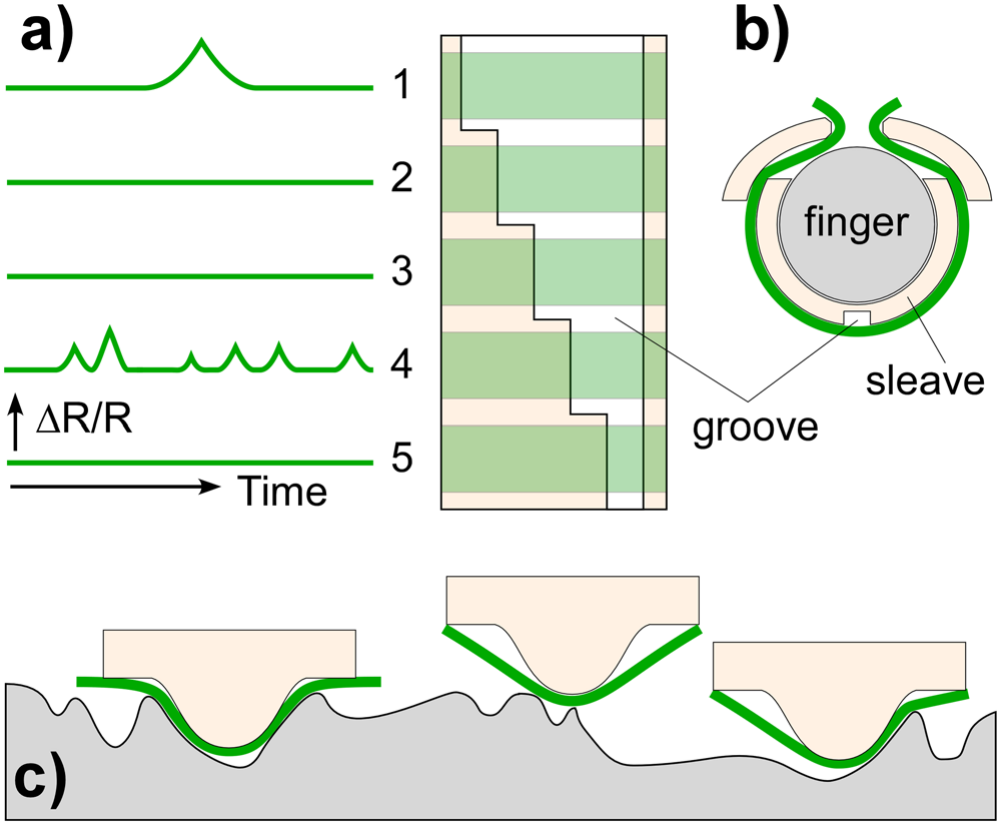
Alternative embodiments. (**a**) Multiple strips over a variable-width groove would provide information concerning surface texture of different sizes. (**b**) The sensor concept in the form of a retrofitable sleeve. (**c**) As a variant configuration, a membrane stretched over a protrusion could also be used to probe surface texture.

After the first large peak, 4, in Fig. 7a there appears to be a second small peak, even though there is no braille dot in that position. This is due to crosstalk over the membrane, as illustrated schematically in the second inset (located in Fig. 7b). First the sensing strip passes over dot 4, producing the main peak when the dot is centered under the groove. The finger then moves past dot 2, a small distance away, which deflects the membrane adjacent to the sensing strip, inducing a smaller strain at the sensor. This artifact could in principle be removed by using a circular hole or divot instead of a groove, or by using a series of independent strips, as illustrated in Fig. 8a.

Figure 7b shows a scan across the lowest row of dots. Dots 7 and 8 are the two most closely spaced dots on the plate at 2.4 mm (Fig. 3a). Because the *x*-direction signal is continuous, taken at 1000 pts/sec, the peaks are clearly resolved. Along this axis, the sensor would readily be able to distinguish dots separated by half that distance, if not better. The change in resistance for the dots in Fig. 7 at different contact depths compared well with our predictions (Supplementary Figure S12), further validating the model.

The spatial resolution between sensing points required for tactile sensing is generally given as 1–2 mm^6^. With a 1.6 mm groove, this approach already exceeds that requirement. The lower limit in feature size will depend on the membrane thickness and groove width, which could be made as small as 10 s of μm with the newest micro-milling machines.

In the *y*-direction, because of the stepping between lines (Fig. 3c), the scan is discrete, as shown in Fig. 7c, so features must be separated by at least 1 mm in order to be resolved. To increase the resolution along *y*, the spacing between the scan lines can be reduced, at the cost of increasing image acquisition time. Using a number of parallel sensors would decrease that time.

## Discussion

The sensor architecture presented in this work is based on displacement rather than force. We had previously established that forces exerted by freestanding membrane sensors are small (<23 mN/mm)^27^. This enables the device to potentially be used to scan profiles of soft surfaces, like foams (500 mN/mm). (If soft biological tissue were being examined, then the membrane stiffness could be reduced.) Furthermore, the design is compact and does not require low-pass filtering in dynamic contact.

Our system bears resemblance to the clever approach taken in ref. 45, in which a single piezoelectric film was draped over a triangular sponge and rubbed once over a series of Braille letters at constant speed. The triangular sponge converted the *y*-position of the dot to a time length of contact, so each letter gave a characteristic signature. The sponge localized the sensing, as was done in our system with the groove. However, we employed scanning in *y* rather than a triangular sensor and physical registration. In extended work by the same group^46^, the sensor was mounted to a finger, and classifiers were applied to compensate for unsteady movements. Another single-element design based on bending a piezoelectric sensor over a braille dot was considered in ref. 50 through simulations. Crosstalk, an issue with tactile sensing, was also investigated.

The question might be asked, what are the advantages of this strip + groove design over a small single-pixel sensor? First, the resolution is better than the cavity size. Second, because small grooves or holes are straightforward to produce, the target size can be readily scaled up or down without changing anything else about the system. Third, mechanical spatial filtering maximizes signals from the desired targets.

Extending the principles demonstrated here suggests two straightforward approaches to probing surface texture across a range of sizes. In one, a series of strips over a groove of increasing width (Fig. 8a), or a set of holes of increasing diameter, would provide a set of profiles corresponding to features of each size, providing a texture analysis in a single sweep. Rastering such a line array over a single feature would provide information about it at multiple length scales. A second sensing approach (Fig. 8c) is based on inverting the geometry used in this work (utilizing another type of ghosting artifact, Supplementary Figure S22) to detect dips rather than protrusions, similar to a profilometer stylus. Fixing the sensing membrane non-conformally over a hillock on the finger, rather than over a groove, would produce a signal when the bump dimensionally matched a surface indentation, making a “fingernail stylus”^51, 52^.

These surface feature sensors could be readily integrated into existing robot fingers that perform manipulations. Adding one or more grooves to a finger is straightforward, and the rubber membrane wrapped around the finger confers grip. Another method of retrofitting is via a sleeve with a groove (or a set of holes or bumps) that can be slid over or snapped onto an existing finger (Fig. 8b).

Practical considerations for evaluating a tactile sensing system should include cost and ease of adaptation^46^. Given the low-cost materials employed and the ease of construction – the system being essentially a carbon-painted rubber band strapped around a part with a small hole in it – the device performed remarkably well.

This paper demonstrates the use of mechanical spatial filtering to enhance the information from a single sensor. Applications of size-specific feature recognition might eventually include tactile assistance to vision-based sensing in unstructured environments when the visual field is complicated, such as picking nuts of only a certain diameter out of a box, or when the object of interest is difficult to see, such as the free end of a piece of tape attached to a roll.

## Conclusions

A simple and inexpensive sensing structure has been presented that uses a profilometry-like method for detecting surface features of a particular size: a stretchable sensor is mounted over a cavity, and the combination is moved over the surface. Features that are smaller than the cavity penetrate into it, causing the sensor to stretch and produce a signal, resulting in “mechanical spatial filtering”; features of matching size result in signal amplification. A model was developed for this new sensing principle in order to guide the design of the finger structure and predict its strain behavior. The approach was demonstrated with braille dots using a piezoresistive sensor, but it can in principle be used with other sensing modalities. Given the simplicity of the structure and its viscoelastic properties, the image quality was noteworthy, since not only were the positions of the braille dots always correctly found, but also their relative heights were revealed, and the shape the of the signal peaks showed that the dots were curved. The ease of implementation should allow this approach to be used on a variety of robot hands, and the sensors treated as disposable.

## Methods

### Fabrication of the sensor membrane

The process for exfoliating graphite has been described previously^27–29^. It begins with expanding acid-intercalated flake graphite (expandable graphite 3772, Asbury Graphite Mills, Inc.) in a microwave oven, resulting in fluffy worm-like tubes. This material (10 g) is sonicated (QSonica, Q700) together with deionized water (1000 g), surfactant (7.5 g, Triton X-100, Sigma-Aldrich), and antifoaming agent (thirty drops, SE-15, Sigma-Aldrich) to produce exfoliated graphite in a suspension. Latex (0.94 g, RD-407, ArtMolds) was blended with the EG suspension (12.2 g) as described in ref. 29. After spray coating and water evaporation, the EG/latex solution leads to a composite containing 25% EG by weight.

Three parallel 1 mm wide sensing strips separated by 2 mm were formed on a latex membrane (Fig. 2b). The pattern was chosen such that the width of the sensing strips roughly matched that of the features to be sensed. In principle, only one sensor is needed. Three sensors were included to permit replicate measurements and in case one failed. They were patterned onto latex sheets by spray-coating the latex/EG solution over a stencil cut from adhesive-backed vinyl sheet film (Craftvinyl blue gloss outdoor vinyl) with a programmable cutting machine (Cricut Explore). The sensing material was applied in 3–5 layers from a distance of 10 cm using a compressed air sprayer (Badger 250–2, 25 psi) until the resistance reached 10–30 kΩ per strip. Each layer was allowed to dry under a heat lamp for 2 minutes before the application of the next layer. After the last layer dried, the latex/EG was wiped from the stencil, to avoid tearing, and the stencil was removed.

Two types of rubber sheets were employed. Those used with the 5-mm wide groove (RubberSheetRoll, latex rubber film S15002, 0.15 mm thick) became gummy over a period of months in laboratory atmosphere. Thus, the brand was changed for the other experiments to one that was stable under storage (ELE International, triaxial rubber membrane 25–7621, 0.3 mm thick).

Sensors that were directly rubbed over the braille plate, as was done with the 5 mm groove, suffered visible damage as a consequence of scanning. For the discrete scans with the 1.6 mm groove, a second sheet was added over the sensing strips to protect them. Sandwiching the sensors provided mechanical protection from surface wear and supported the carbon fiber connections. The latex sheets adhered to each other, and the incompletely dried latex/EG solution at the contact pads further served as a glue. (Because of the limited permeability of the latex sheets, if the glue layer was too wet, then water was trapped, creating a conducting path around the sensor and shorting the three sensors together). The second sheet also provides electrical isolation, although this was not an issue in these measurements.

The sensor used for the continuous scans with the 1.6 mm groove was not protected with a second latex sheet but was instead sprayed with a thin latex coating and mounted with the sensing material facing the finger rather than the target, which provided similar protection from surface wear. Because the printed finger surface was somewhat rough, a layer of relatively smooth painter’s tape was applied to it (Fig. 2a) to minimize potential damage by rubbing.

The sensing signal is influenced by variation among the handmade sensors, prior use, aging of the membrane, and shifting of the seating of the membrane over the finger. However, the repeatability of these sensors for braille reading was still 100%, with accurate imaging in all tests.

### Electrical connections

Electrical leads were created by attaching a length of 22 AWG wire to two tows of carbon microfibers from a carbon fiber braid (Toray T300) having + /−45° fiber orientations. Each tow consisted of approximately 10^3^ carbon fibers and had a weight of 7.8 g/100 m, yielding a calculated cross-sectional area of 0.0385 mm^2^ for each tow. The conductivity of the carbon fiber is 1.7 × 10^−3^ S/cm, 10^3^ times lower than that of copper. Since only a short length was used, the resistance drops were no more than a few Ohms, orders of magnitude less than the sensor resistance. The insulation was stripped from a 2 cm length at the end of the wire, the carbon fiber was placed alongside the wire, and the two were connected with a length of 1/8″ diameter heat shrink tubing. For the continuous scans (Fig. 6), the sensor was fabricated in a simpler way: the sensing strip was extended beyond the clamps, and alligator clips were used instead of the carbon fiber connectors. This allowed the membrane to be clamped onto the finger prior to making electrical connections.

The carbon microfiber leads were connected to the ends of the sensing strips with the same EG/latex solution used in the sensing layer (Fig. 2c). The ends of the tows were manually spread out, placed on the contact pads, and wetted with the solution. To ensure that the carbon fiber tows did not move during this process, the leads were temporarily taped in place. Droplets of EG/latex were added over the contact pad and dried; typically 3–5 droplets produced a high-strength mechanical and high-conductivity electrical connection. The leads were, however, prone to peeling off the surface under torque, because of their greater stiffness.

Later sensors were also made without integrated leads, instead utilizing alligator clips clamped directly onto the ends of the sensing strips. In order to extend the sensing material to the edges of the latex sheets, the stencils were augmented with painter’s tape. This simplified prototype production, allowed easier connection of electrical leads, and it decreased the amount of care required in handling the sensors.

### Test rig and touch protocol

Each sensing strip was put into a voltage divider circuit with a 30 kΩ fixed resistor, over which 5 V was applied (Korad KA3005 P 30 V, 5 A programmable power supply). The voltage over the sensor was obtained with a National Instruments DAQ (NI USB-6008 Multifunction IO) and converted to resistance in software.

The braille targets consisted of a pattern of bumps 2 mm in diameter punched into a brushed stainless steel sheet (Figs 2d and 3a). These coupons were glued to a Delrin base, which had holes for bolts (Fig. 2d). Dot heights were measured with a digital depth micrometer (Mitutoyo, 0.00005” 313703), 5 times each, and averaged.

The target and the finger were mounted on a motorized test bed (Fig. 2e,g) consisting of an *x-y* positioning table comprising two worm-driven slides (Velmex XN10-0060-M01-71) driven by stepper motors (Velmex PK245-01AA) and a vertical *z*-axis stepper-driven actuator (PI M-229.26S). The motion of the test rig was automatically controlled using an Arduino microprocessor running custom software. (The code is provided as Electronic Supplementary Information). The braille target was mounted to the *x*-*y* table, and the finger was mounted to the vertical *z* actuator. The *x*, *y*, and *z* actuators were stepper motors driven with open-loop control. To ensure positioning accuracy, the *z*-axis actuator was zeroed after every touch using a Hall effect limit switch. Because testing showed that the *x*-*y* table actuators did not lose an appreciable number of steps even after extended operation, they were later simply returned to the starting position with open-loop control between tests. The vertical actuator was suspended on a Delrin frame that had some flexibility.

The finger scanned a 33 × 12.5 mm^2^ area of the braille plate encompassing all four of the braille figures. For the discrete case, the finger was brought into contact with the surface on a 0.5 mm grid (Fig. 3c), giving 3–4 points along each braille dot. A single touch consisted of lowering the finger to a fixed distance at a fixed velocity followed by retraction at the same velocity; each touch took 15 seconds, and resetting to the beginning of the next row took 100 seconds. The scan was accomplished line-by-line, right to left. The entire scan took 7 hours. For the continuous scans, the area was continuously swept line-by-line. Each line was swiped twice – forward and backwards – before moving on to the next line, producing two images.

Images were generated in 45 minutes, an order of magnitude faster than it took to produce the discrete images. This was not a limit of the sensor, but of the stepper motors that were used in the test rig, which were run at their maximum operating speed (approx. 30 mm/min). It would be reasonable to expect that a dexterous robot hand could rub a surface much more quickly.

### Data Processing

Time-series voltage data were collected at a 500 Hz sample rate with LabView Signal Express and preprocessed with a low-pass filter (2^nd^ order Butterworth, cutoff frequency 10 Hz). The peak voltage during each touch of the plate was stored in a 66-by-25 array. A baseline voltage was taken before the first touch of the scan, and the baseline was subtracted from the peak voltage. The same baseline was used for the entire image, as discussed in the Supplementary Information (Supplementary Figures S14–19). The adjusted voltages were converted to resistance and then normalized by the baseline resistance to yield ΔR/R. Images were created using R^53–55^, a statistical programming language.

## Electronic supplementary material

Supplementary Information
