# Supplementary material for: Targeted Feature Recognition Using Mechanical Spatial Filtering with a Low-Cost Compliant Strain Sensor
Source: Sci Rep. 2017 Jul 11;7:5118. doi: 10.1038/s41598-017-05341-w (PMC5506026; doi:10.1038/s41598-017-05341-w)
Supplement: Supplementary file 1 — Supplementary Information [file 41598_2017_5341_MOESM1_ESM.pdf]

Supplementary Information
for

Targeted Feature Recognition Using Mechanical Spatial Filtering with a Low-Cost Compliant Strain Sensor

Eli M. Barnett, Julian J. Lofton, Miao Yu, Hugh Bruck, and Elisabeth Smela
University of Maryland

Modeling

In this section, the strain as a function of notch displacement for three shapes is modeled. The triangle example goes through the mathematical model steps of the paper in detail, and then shows a simple geometric approach that gives the same result. This is followed by a hemisphere example in which the parametric equation for the mathematical approach is provided, and then the geometric approach is detailed. Finally, the strain profile is derived for a rectangular feature.

Triangle Example

The example of a triangle is used to demonstrate the modeling approach in the "Modeling contact" section of the paper because of its mathematical and conceptual simplicity, which allows focus on the method (Supplementary Figure S1). The triangle has height h and width $2h$, so $x_{max} = h$. The notch is wider and taller than the feature (case 1), $b = 3h$. The position of the notch is chosen to be $u = h/2$.

Mathematical model.

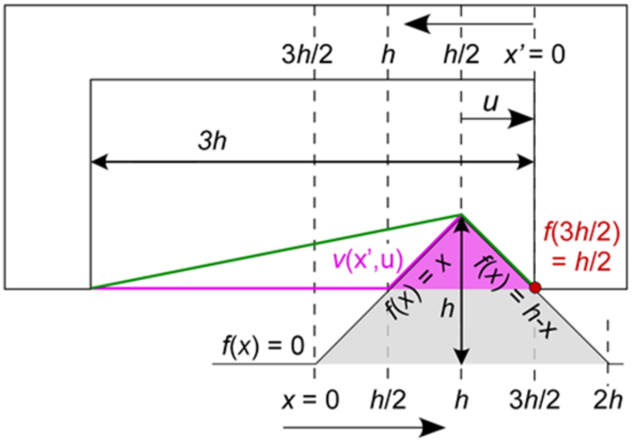

**Supplementary Figure S1. Triangle example with $u = h/2$. **

The triangle is defined piece-wise:

(1) $f(x) = 0$ for $x < 0$
$f(x) = x$ for $0 < x < h$

$f(x) = h - x$ for $h < x < 2h$
$f(x) = 0$ for $x > 2h$.

First find the displacement field v. Beyond the triangle's apex, for $0 < x' < h/2$ (or $h < x < 3h/2$),

(2)
$$v(x',u) = f(u + x_{max} - x') - f(u + x_{max})$$
$$v\left(x',\frac{h}{2}\right) = f\left(\frac{h}{2} + h - x'\right) - f\left(\frac{h}{2} + h\right) = f\left(\frac{3h}{2} - x'\right) - f\left(\frac{3h}{2}\right).$$

Since $f(x) = h - x$ here,

(3)
$$v\left(x',\frac{h}{2}\right) = \left(h - \frac{3h}{2} + x'\right) - \left(h - \frac{3h}{2}\right) = x'.$$

So v increases from 0 to $h/2$, as is evident from the figure. The value of the reference point $f(u + x_{max})$ is $h/2$. To the left of the apex, for $h/2 < x' < 3h/2$ (or $0 < x < h$), where $f(x) = x$

(4)
$$v\left(x',\frac{h}{2}\right) = f\left(\frac{3h}{2} - x'\right) - \frac{h}{2} = \frac{3h}{2} - x' - \frac{h}{2} = h - x'$$

which is greater than 0 only for $h/2 < x' < h$. For $3h/2 < x'$, $v(x',h/2) = 0$.

Next any take-off points are found. To the right of x_{max}, this would occur if

(5)
$$\frac{df(u + x_{max} - x')}{dx} \le -\frac{v(x',u)}{x'}.$$

Just above $x' = h/2$ (at $x = h + \delta$), the slope of the triangle is

(6)
$$\frac{df(h+\delta)}{dx} = \frac{d(h-x)}{dx} = -1,$$

and the slope of the straight line from the apex to the corner is

(7)
$$-\frac{v(x',u)}{x'} = -\frac{x'}{x'} = -\frac{0.5h}{0.5h} = -1.$$

Since the former is not less than the latter, the membrane follows the surface. To the left of x_{max}, the condition is instead

(8)
$$\frac{df(u + x_{max} - x')}{dx} \ge \frac{v(x',u)}{b - x'}.$$

The slope of the triangle feature there is then

(9)
$$\frac{df(h-\delta)}{dx} = \frac{d(x)}{dx} = 1,$$

while the slope of the straight line from the apex to the corner is given by

(10)
$$\frac{v(x',u)}{b - x'} = \frac{h - x'}{3h - x'} = \frac{h - 0.5h}{3h - 0.5h} = \frac{0.5h}{2.5h} = 0.2 < 1.$$

The condition for take-off is met at the apex, $p_1 = h$, so the membrane leaves the surface.

Next find the total deformed length of the membrane, given in general by by:

(11) $$L_d(u) = \int_0^b \sqrt{1 + \left(\frac{dv(x',u)}{dx'}\right)^2} dx'$$

For this case, to the right of the apex v is given by equation (3), and to the left of the apex

(12) $$v(x',u) = \frac{b - x'}{p_1 - (u + x_{\text{max}} - b)}\left[f(p_1) - f(u + x_{\text{max}})\right] = \frac{3h - x'}{h - \left(\frac{3h}{2} - 3h\right)}\left[h - \frac{h}{2}\right] = \frac{3h - x'}{\frac{5h}{2}}\frac{h}{2} = \frac{3h - x'}{5},$$

which drops from $h/2$ at $x' = h/2$ to 0 at $x' = 3h$, as shown in the figure. Plugging this into the integral:

$$L_d\left(\frac{3h}{2}\right) = \int_0^{h/2} \sqrt{1 + \left(\frac{dx'}{dx'}\right)^2} dx' + \int_{h/2}^{3h} \sqrt{1 + \left(\frac{d\left(\frac{3h - x'}{5}\right)}{dx'}\right)^2} dx'$$

(13) $$= \int_0^{h/2} \sqrt{1 + (1)^2} dx' + \int_{h/2}^{3h} \sqrt{1 + \left(-\frac{1}{5}\right)^2} dx' = \sqrt{2}\left(\frac{h}{2} - 0\right) + \sqrt{\frac{26}{25}}\left(3h - \frac{h}{2}\right).$$

$$= \frac{h}{2}\sqrt{2} + \sqrt{\frac{26}{25}}\left(5\frac{h}{2}\right) = \frac{h}{2}\left(\sqrt{2} + \sqrt{26}\right) = \frac{h}{2}(1.41 + 5.1) = 3.26h$$

Within the notch, this corresponds to a strain of 0.26/3.0 = 8.6%.

Geometric model.

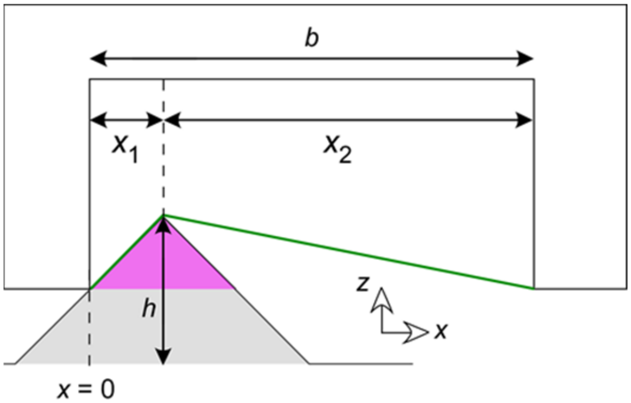

Supplementary Figure S2. Triangle example with $x_1 = h/2$, $b = 3h$.

It is often simpler to find the strain using a geometric model. The same triangle is used here as in the previous section, but for convenience we define an x-axis variable x_1 with $x_1 = 0$ at the left inner edge of the notch (Supplementary Figure S2).

Considering penetration of the triangle into the left half of the notch, the tip penetrates to a maximum of

(14) $z = u$ for $u < h$
$z = h$ for $u \geq h$

The deformed membrane length is thus given by

$$(15) \quad L_d = \sqrt{x_1^2 + z^2} + \sqrt{x_2^2 + z^2} = \sqrt{x_1^2 + z^2} + \sqrt{(b - x_1)^2 + z^2} \ ,$$

where x_1 is the distance of the probe tip from the left edge and $x_2 = b - x_1$. When $x_1 = h/2$ and substituting $b = 3h$,

$$(16) \quad L_d = \sqrt{\left(\frac{h}{2}\right)^2 + \left(\frac{h}{2}\right)^2} + \sqrt{\left(3h - \frac{h}{2}\right)^2 + \left(\frac{h}{2}\right)^2} = \frac{h}{2}\left(\sqrt{2} + \sqrt{26}\right)$$

This is the same result as found in equation (13). To find the strain on the right side of the notch, the results are mirrored by exchanging the definitions of x_1 and x_2.

Groove dimensions.

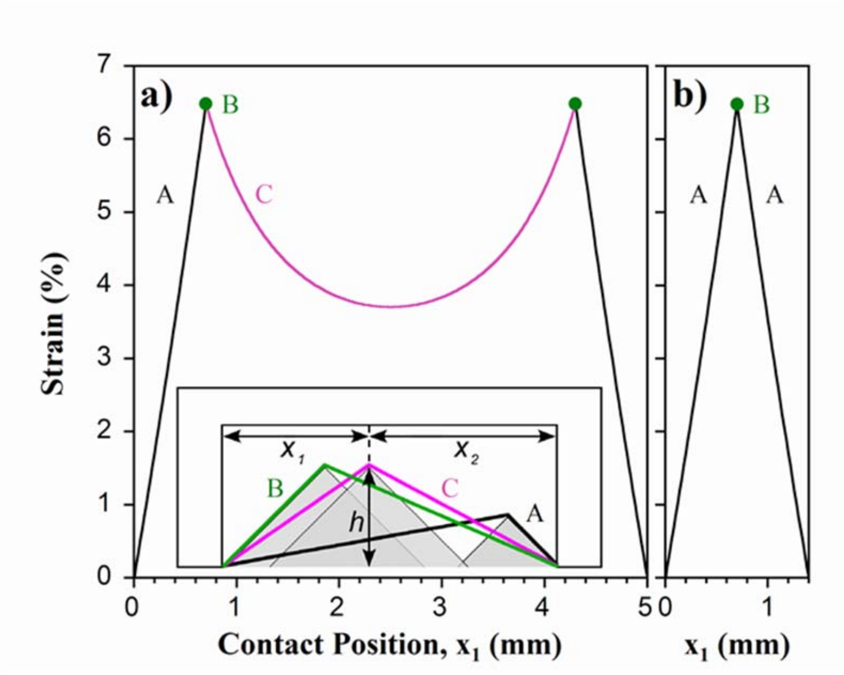

Supplementary Figure S3. a) Predicted variation of sensor strain with position of a triangular indenter with a height *h* = 0.7 mm under a *b* = 5 mm wide groove. The stretched length of the membrane, *L*, is given by the lengths of the two linear segments, illustrated in the inset. At the edges of the groove (A), the indentation increases linearly (equation (14)), resulting in linearly increasing strain. At point (B), the indentation reaches its maximum extent, *h*. With greater distances from the edge (C), *L* decreases. b) Variation in strain for a groove 1.4 mm wide, matching the triangle width.

To gain an intuitive understanding for the presence of the ghost artifacts in the image shown in Figure 4b of the paper, the geometric model was used to evaluate the strain created by a

triangular feature. In the experiments the braille dots were 0.7 mm tall, so this value was used for *h* here. The model was evaluated for values of x_1 between 0 and *b*. The first groove was wider and taller than the feature, with a width of *b* = 5 mm. Supplementary Figure S3a shows the strain as a function of contact position. The strain increases until $x_1 = h$ and then decreases again as the feature moves beyond that point towards the center.

The *x*-positions of the peaks are related to the width of the surface feature. For the triangle, the peaks occur at a distance *h* from the two edges, when the triangle first (or last) fully indents the membrane. A wider object would therefore result in more closely spaced peaks, reaching the limit of a single peak when the feature size matches the groove, i.e. when $2h = b$ in this example, as shown in Supplementary Figure S3b. The distance to the ghost signal can therefore in principle be used to determine the size of an object smaller than the groove. The peak amplitudes remain unchanged until the feature becomes wider than the notch, when the amplitude decreases.

<u>Hemisphere Example</u>

The tops of the braille dots can be approximated as hemispherical, with a half-circle cross-section.

Mathematical model.

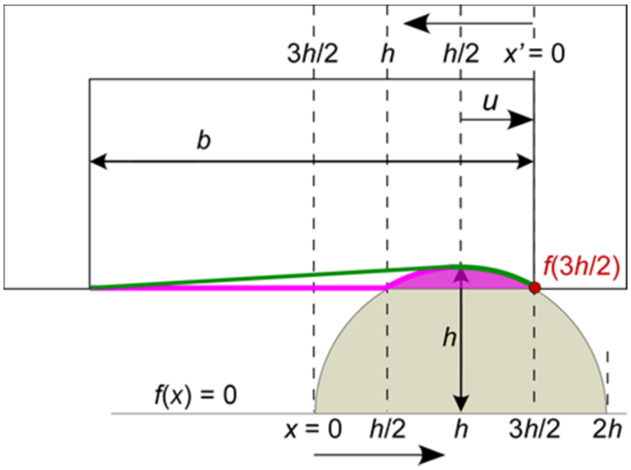

Supplementary Figure S4. Semi-circle example with *r* = *h* = 0.7, *b* = 5, *u* = *h*/2.

Choose u = r/2. To calculate the strain using the mathematical model, the same procedure would be followed as above but using a parametric equation for a circle, putting $x = 0$ at its left edge:

(17) $f(x) = 0$ for $x < 0$

$$f(x) = \sqrt{r^2 - (r - x)^2} \quad \text{for } 0 < x < 2r$$

$f(x) = 0$ for $x > 2r$

Find the displacement field v.

$$v(x',u) = f(u + x_{max} - x') - f(u + x_{max})$$

$$v\left(x',\frac{r}{2}\right) = f\left(\frac{r}{2}+r-x'\right) - f\left(\frac{r}{2}+r\right) = f\left(\frac{3r}{2}-x'\right) - f\left(\frac{3r}{2}\right)$$

(18) $$= \sqrt{r^2 - \left[r - \left(\frac{3r}{2}-x'\right)\right]^2} - \sqrt{r^2 - \left[r-\left(\frac{3r}{2}\right)\right]^2} .$$

$$= \sqrt{r^2 - \left[\left(-\frac{r}{2}+x'\right)\right]^2} - \sqrt{r^2-\frac{r^2}{4}} = \sqrt{r^2 - \left[\frac{r^2}{4} - rx' + x'^2\right]} - \sqrt{\frac{3r^2}{4}}$$

$$= \sqrt{\frac{3r^2}{4}+rx'-x'^2} - \sqrt{\frac{3r^2}{4}} = \sqrt{0.3675+0.7x'-x'^2} - 0.6062$$

This is greater than 0 for *0 < x' <* h/2, as shown in Supplementary Figure S5 by the red line, found numerically.

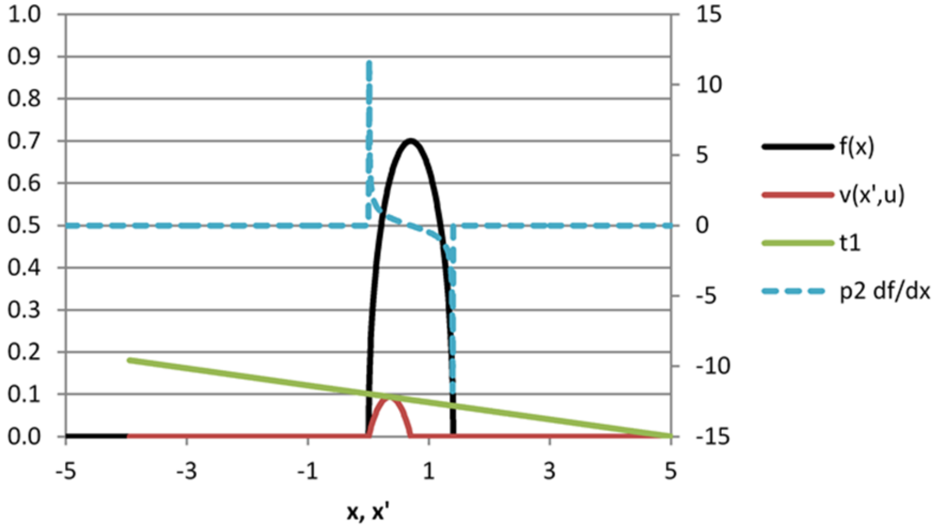

Supplementary Figure S5. Mathematical mode for semi-circle example with *r* = *h* = 0.7, *b* = 3*h*, *u* = *h*/2. Note that *x'* is shown on the same *x*-axis.

Find the take-off points and connecting lines. To the right of x_{max}, by inspection of Supplementary Figure S4, there is no p_2. This is confirmed comparing the slope of f(x) to the slope of a line from the point to the notch inner corner. To the left of x_{max}, we need to find p_1 by comparing the local slope of the function with the connecting line:

(19) $$\frac{df(u + x_{max} - x')}{dx} \geq \frac{v(x',u)}{b - x'} .$$

This was found numerically to be at the top of the semi-circle. The slope is then given by

(20) $$v(x',u) = \frac{b-x'}{p_1 - (u + x_{max} - b)}\left[f(p_1) - f(u + x_{max})\right]$$

as shown by the green line in Supplementary Figure S4.

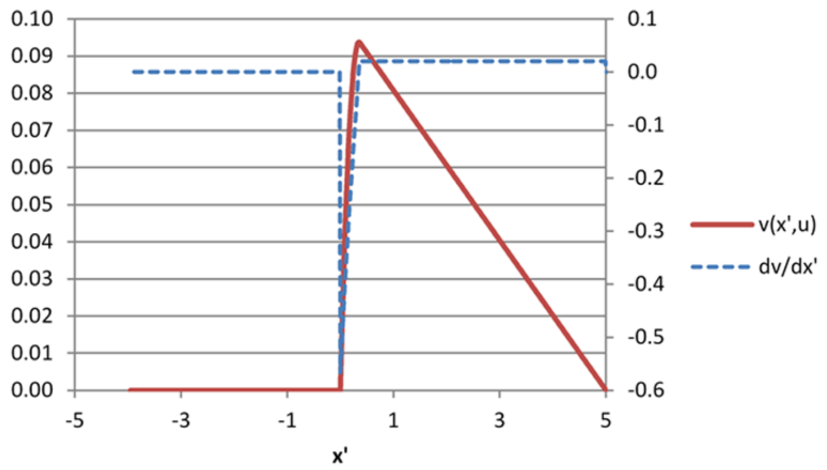

Supplementary Figure S6. Mathematical mode for semi-circle example with *r* = *h* = 0.7, *b* = 3*h*, *u* = *h*/2. The final v(x',u) is shown, along with its derivative.

Find the length of v(x',u) and the strain. The length is found by integrating. Here L_{ex} is taken as zero. The $v(x',u)$ used in this calculation is shown by the red line in Supplementary Figure S6, and its slope is shown by the dashed blue line.

(21) $$L_d(u) = \int_0^b \sqrt{1 + \left(\frac{dv(x',u)}{dx'}\right)^2}\, dx' + L_{ex}$$.

The strain is found to be 0.35%. To map out the strain as a function of u, this process is repeated for each u. The three segments of $v(x',u)$ for two u = 1 and u = 2 are shown in Supplementary Figure S7. The strains in these two cases were 6.37% and 4.12%, respectively.

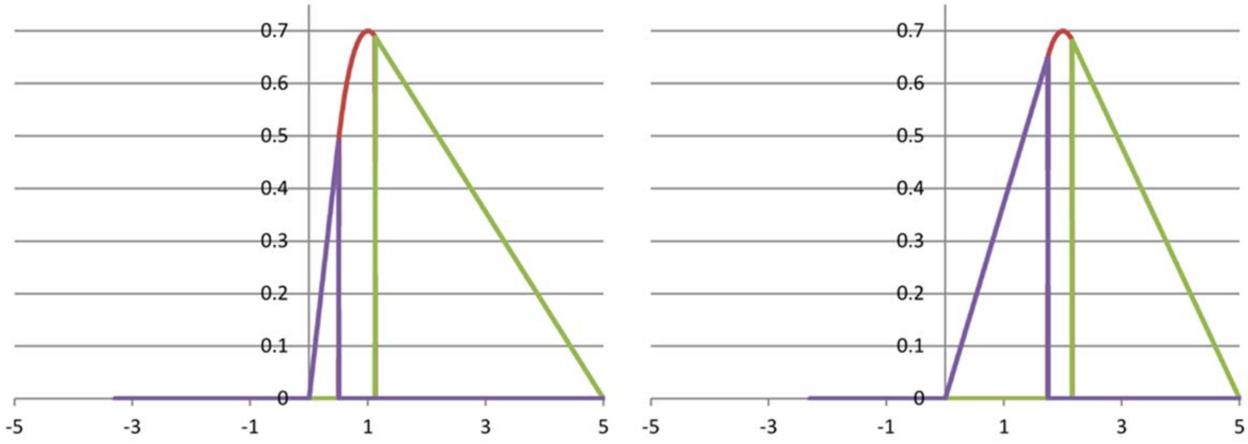

Supplementary Figure S7. Mathematical mode for semi-circle example with *r* = *h* = 0.7, *b* = 3*h*, and *u* = 1 and *u* = 2 showing how the connecting lines (purple, green) and contact line (red) change. .

Geometric model.

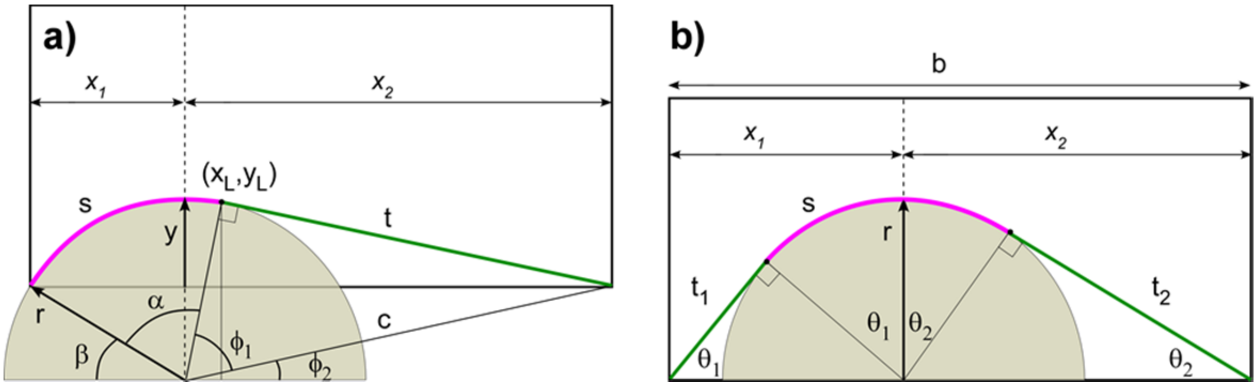

Supplementary Figure S8. a) Geometric model for a circular cross section as the feature begins to penetrate into the notch and deflect the membrane. b) When the feature has penetrated its full height into the notch.

A geometric model (Supplementary Figure S8) was used to find the strain as a function of *u* because of the relative simplicity of this approach. For the case that $x_1 \leq r$, when the feature only partially penetrates into the notch, the following relationships hold true.

(22) $$\beta = \cos^{-1}\left(\frac{x_1}{r}\right)$$

(23) $$\varphi_2 = \tan^{-1}\frac{r\sin\beta}{x_2} = \tan^{-1}\frac{r\sin\beta}{b-x_1}$$

(24) $$c = \sqrt{(r\sin\beta)^2 + x_2^2} = \sqrt{t^2 + r^2}$$
$$t = \sqrt{(r\sin\beta)^2 + x_2^2 - r^2} = \sqrt{(r\sin\beta)^2 + (b-x_1)^2 - r^2}$$

(25) $$\varphi_1 = \tan^{-1}\frac{t}{r}$$

(26) $$\alpha = 180 - \beta - \varphi_1 - \varphi_2$$

(27) $$s = r\alpha$$

(28) $$L_d = s + t$$

When $r < x_1 \leq b/2$, the feature penetrates to its maximum height into the notch, and these relationships apply instead.

(29) $$t_1 = \sqrt{x_1^2 - r^2}$$
$$t_2 = \sqrt{x_2^2 - r^2} = \sqrt{(b-x_1)^2 - r^2}$$

(30) $$\theta_1 = \tan^{-1}\frac{r}{t_1}$$
$$\theta_2 = \tan^{-1}\frac{r}{t_2}$$

(31) $$s = r\left(\theta_1 + \theta_2\right)$$

(32) $$L_d = s + t_1 + t_2$$

On the right hand side of the notch, the above results are mirrored by exchanging x_1 and x_2. The results for several values of *b* are shown in Figure 5 of the paper. The results of the geometric and mathematical models are compared in Supplementary Figure S9.

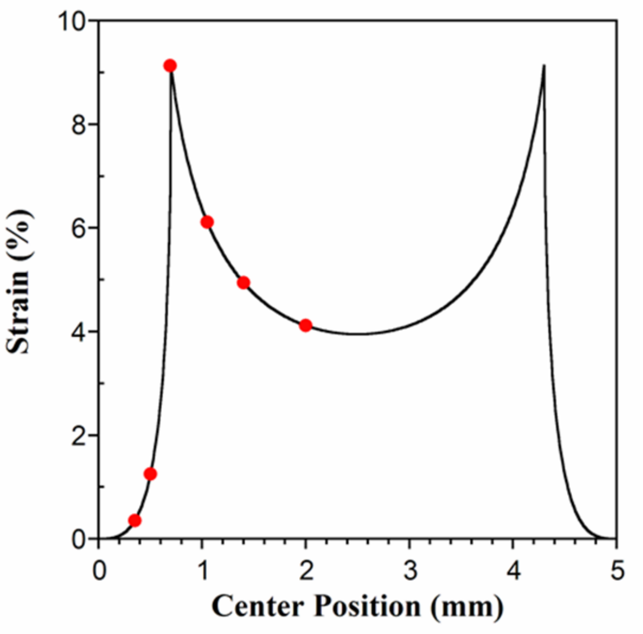

Supplementary Figure S9. Comparison of the geometric (black line) and mathematical (red points) models for *r* = 0.7, *b* = 5.

Intuitive explanation.

Assuming that the finger is in continuous contact with the target surface (the experiments used a fixed vertical offset from the surface), as its *x*-position varies, so does the part interacting with the surface. In some positions, the bottom-most tip of the probe touches (Supplementary Figure S10a, pink cases 1 and 3), at other times the rounded edge does (blue case 2), and when the probe is over a small feature, the membrane is in contact and is deflected (green case 4). The probe vertical position therefore changes, as indicated by the bars in Supplementary Figure S10b, which are color-coded to indicate the cases. The regions that produce a change in resistance (green bars) are boxed. If the signal is proportional to the membrane deflection, neglecting secondary effects, then its appearance will be as shown in Supplementary Figure S10c. This agrees with the shapes of the peaks shown in Figure 5d,e in the paper.

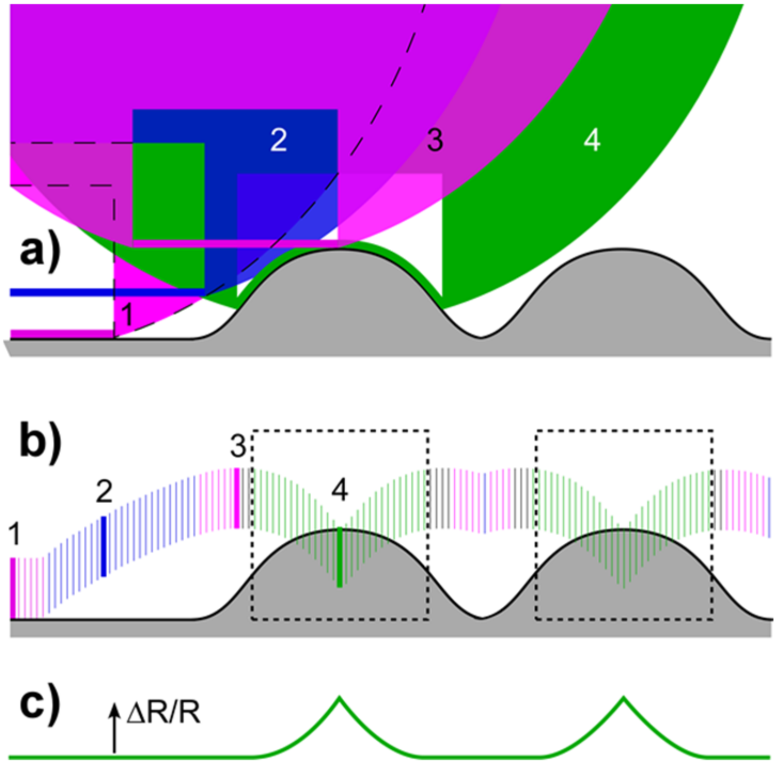

Supplementary Figure S10. Designing a sensor for spatial filtering. a) The finger is in contact with the surface at various *x*-positions, touching at: the tip (pink cases 1 and 3), side (blue, 2), or membrane (green, 4). b) Vertical positions are represented by bars, and the part of the sensor in contact by color. Heavy bars correspond to the positions shown in (a). Signal-producing regions (green) are boxed. c) A signal proportional to membrane deflection.

Nonlinear Amplification

The experiments used a constant offset from the surface, which is equivalent to case 3 (notch less deep than the feature height). To show the effect of increasing the depth of contact (as was done in Figure 6b-d), the model was run at various vertical offsets (i.e., limiting *y* in Supplementary Figure S8a to a maximum penetration depth y_{max}) for a semi-circular feature of $r = 0.7$. The strain profiles are shown in Supplementary Figure S11. A curve fit to the peak strains is shown in Supplementary Figure S12. A quadratic expression fit these points well ($R^2 = 0.9999$). This nonlinear dependence of strain on maximum penetration depth is another manifestation of the amplification caused by spatial filtering.

Also shown in Supplementary Figure S12 are strains obtained from the experimental data of Figure 6b-d and Figure 7 at constant offsets of 100 μm (0.1 mm). The peak values for ΔR/R were divided by the gauge factor, GF = 14, to yield the strain. The unknown initial offset was chosen to bring the data onto the curve, using 0.17 mm for the tallest peaks 4 & 5, 0.12 mm for the medium-size peaks 6-8, and 0.07 mm for the smallest peak 7. These offsets of 50 μm are consistent with differences in actual dot heights on these braille coupons. *Not only is the expected quadratic dependence seen, but also the experimental strain amplitudes are consistent with the model predictions.*

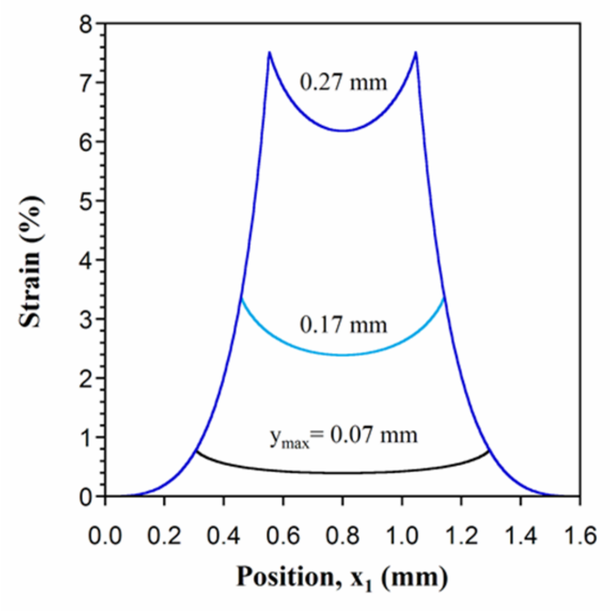

Supplementary Figure S11. Strain versus position in the notch (*b* = 1.6 mm) for a semi-circle of *r* = 0.7 mm for values of y_{max} differing by 0.1 mm. The peak strain increases nonlinearly.

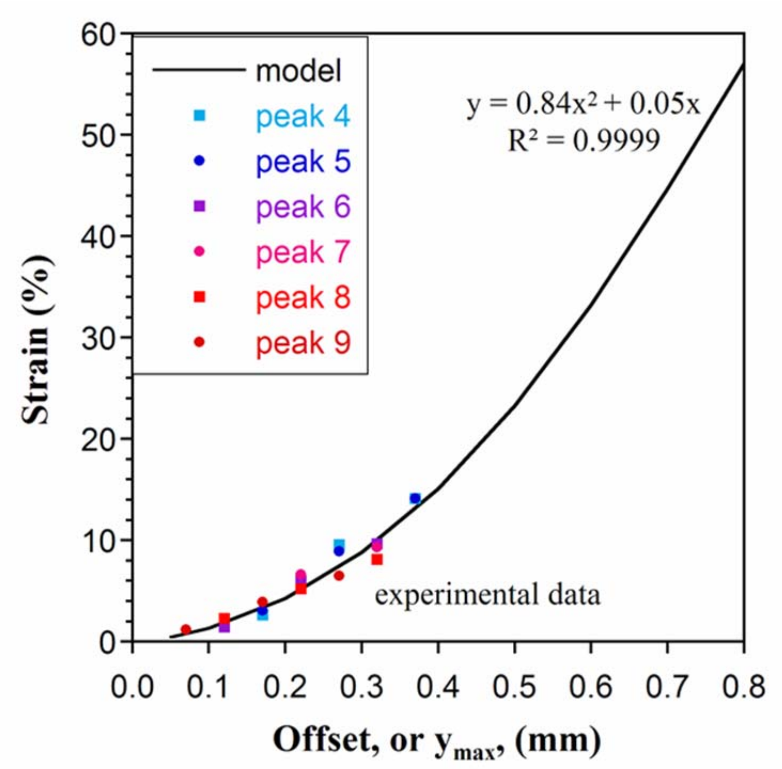

Supplementary Figure S12. Strain as a function of maximum penetration depth, or constant offset from the surface for a semi-circle of *r* = 0.7 mm and notch *b* = 1.6 mm. The solid line shows the peak strain predicted by the models (both geometric and mathematical), and the points show the strain found in the experiments, converted from the signal using the gauge factor.

Rectangle Example

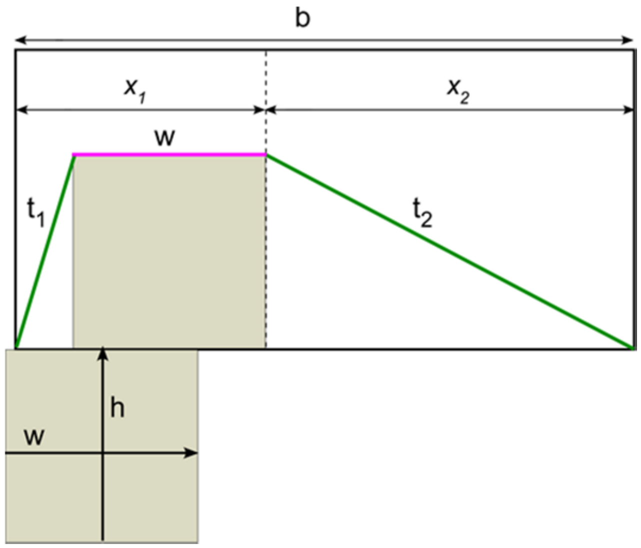

Supplementary Figure S13. Geometric model for a rectangular cross section. Note that the definition of x_1 differs here from the previous cases; the results can be aligned by shifting the *x*-axis by *w*/2.

For a rectangle of height *h* and width *w*, the feature goes abruptly from being outside the notch (no strain, $L_d = b$) to entirely within the notch. The geometric model (Supplementary Figure S13) is:

(33)
$$t_1 = \sqrt{h^2 + (x_1 - w)^2}$$
$$t_2 = \sqrt{h^2 + (b - x_1)^2}$$

(34)
$$L_d = w + t_1 + t_2$$

The results are shown in Figure 5d of the paper.

Time Course Data for a Single Touch

Data for a single touch are shown in Supplementary Figure S14. There is a baseline voltage before the touch, an immediate rise in voltage when the membrane is strained by the braille dot (corresponding to an increase in resistance *R*), followed by a graduate drop that does not return all the way to the baseline due to viscoelastic effects.

To form the images in the main text, the baseline voltage before the first touch of the scan was subtracted from the peak voltage. The adjusted voltages were converted to resistance and then normalized by the baseline resistance to yield $\Delta R/R$.

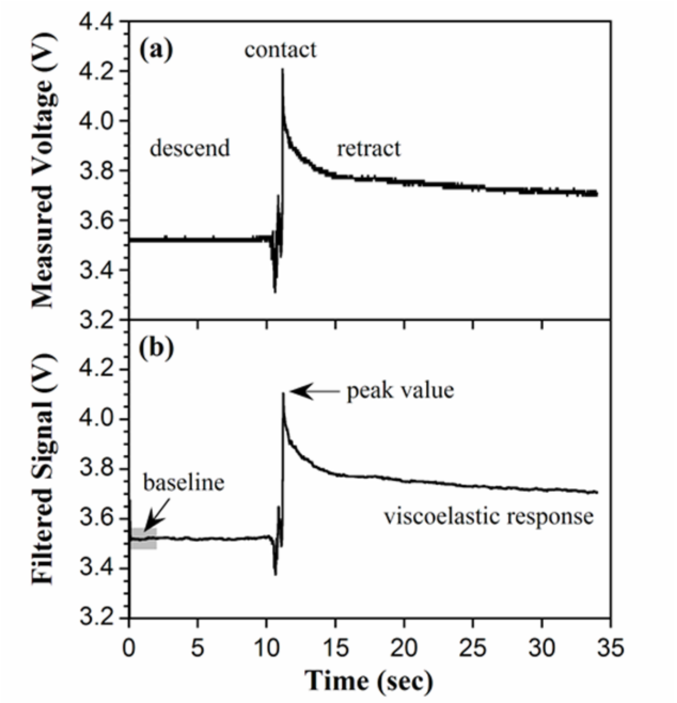

Supplementary Figure S14. a) The raw voltage measured over the sensor in a voltage divider circuit. b) The signal after low-pass filtering, showing the peak value used in the image, prior to baseline correction.

Initial Characterization with an Indenter

Before attempting braille scans, the sensor was characterized by bringing the finger (groove-with-foam variant) repeatedly into contact with a 0.25’’ (6.4 mm) diameter wooden dowel (the “indenter”). The finger was positioned to center a single sensing strip above the indenter. The sensor was brought into contact sequentially at three different depths: 0.5 mm, 1 mm, and 1.5 mm, measured from the lowest finger position at which the sensor generated no signal. Touches were separated by 10 seconds, and the series of three touches was repeated 40 times.

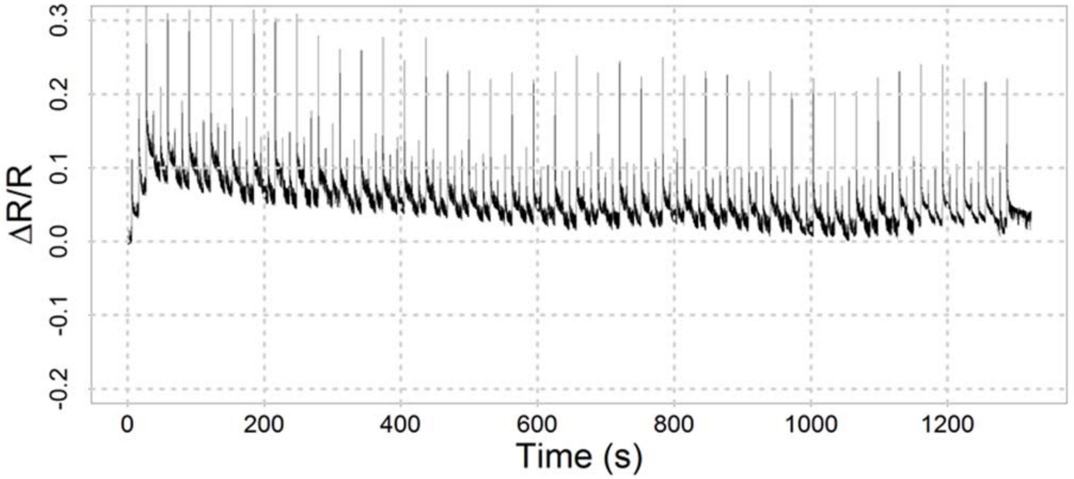

Supplementary Figure S15. Time-series change in resistance of a sensor during indenting testing, normalized by the initial (no-contact) resistance.

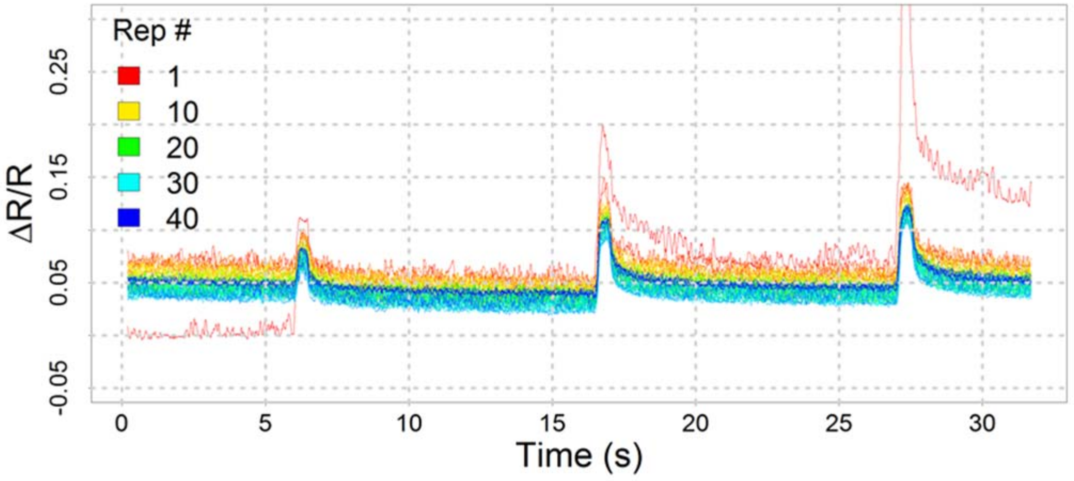

Supplementary Figure S16. Time-series data from Supplementary Figure S15, partitioned and colored by repetition number.

Supplementary Figure S15 and Supplementary Figure S16 show the response of the sensor over the course of the test. In Supplementary Figure S15, the response is plotted versus time over the duration of the test. In Supplementary Figure S16, the time-series data are partitioned by sets of three touches, with the repetition number indicated by color. Apparent in both is a

conditioning effect: the signal decreased as the test progressed. This effect may have been due to the mechanical properties of the foam substrate or the latex skin or both.

In an attempt to correct for the conditioning effect, the baseline resistance at the start of each repetition was subtracted, as shown in Supplementary Figure S17. The conditioning effect was mostly removed. There remained a large drop after the first set of touches, but the decay between subsequent touches was much smaller.

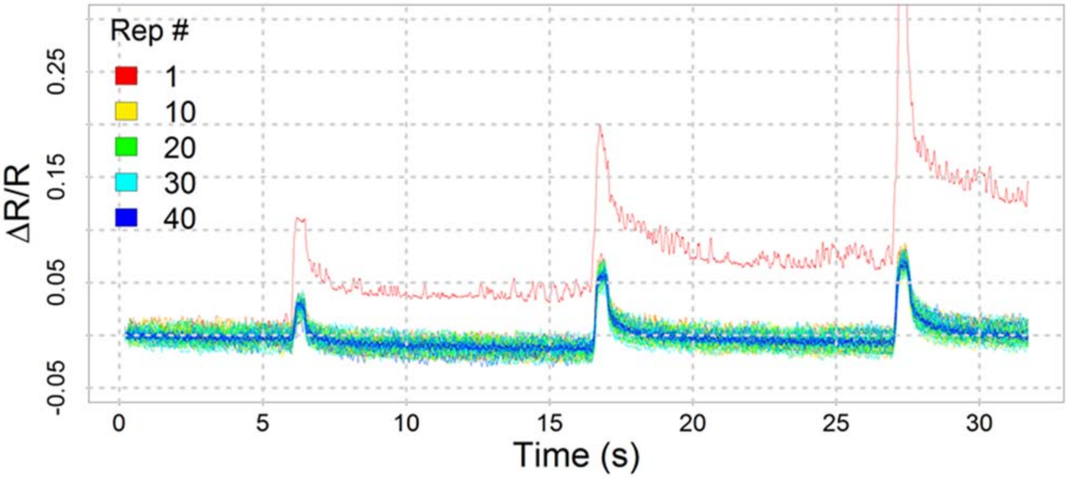

Supplementary Figure S17. Time-series data from Supplementary Figure S15, partitioned and colored by repetition, with the baseline for each repetition subtracted.

The signal in these figures contained notably more noise than the figures in the main paper. This was due to the use of a different power supply (Hantek PPS2116A), which became noisy when it was left on for extended periods.

Zeroing Between Bumps

Based on the indenter tests, the baseline between touches was subtracted from the discrete braille scan. The results, processed from the raw data in Figure 4b, are shown in Supplementary Figure S18.

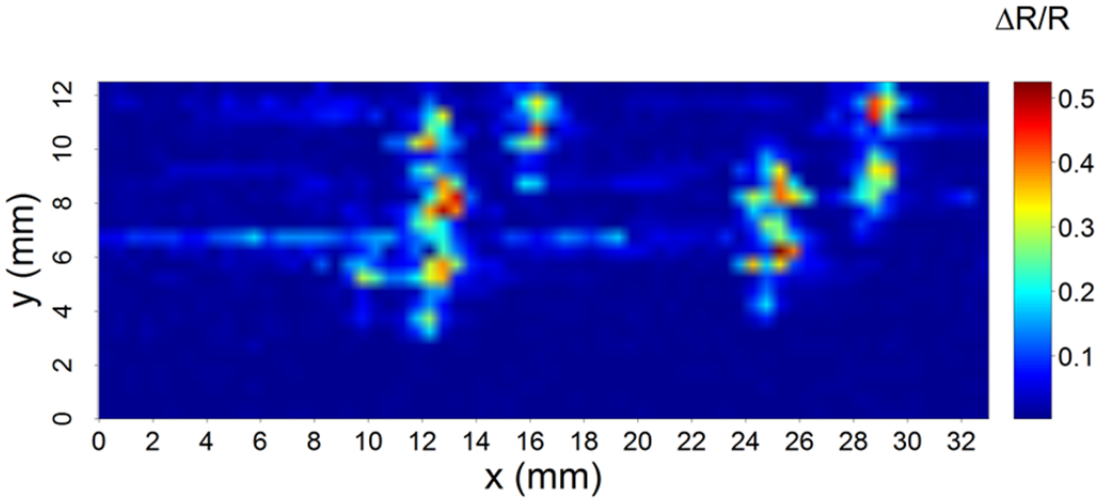

Supplementary Figure S18. Normalized change in resistance from the baseline before each touch.

The braille pattern was more evident when the baseline was taken only once at the beginning of the scan than before each touch. This was contrary to our expectation. Some key differences might explain this discrepancy. Firstly, the design of the finger was changed between the indenter tests and the braille scan: the foam was removed and the groove was narrowed. Secondly, the 6.4 mm wooden dowel was larger than the 1.5 mm braille bump.

At first glance the image appears noisy, with sharp peaks in places that do not seem to correspond with the braille bumps. However, the pattern was consistently repeated in four independent scans (not shown). It is possible that the sharp points in Supplementary Figure S18 occurred because, since the grid of points used to scan the braille plate does not coincide with the braille geometry, at some points the braille bump lies more cleanly within the groove of the finger than at others.

In an attempt to recover the overall shape of the braille plate from the moving-baseline data, they were convolved with a 2D Gaussian filter with σ roughly corresponding to the size of a braille bump. The result is shown in Supplementary Figure S19. The image improved, but was still inferior to the unprocessed image.

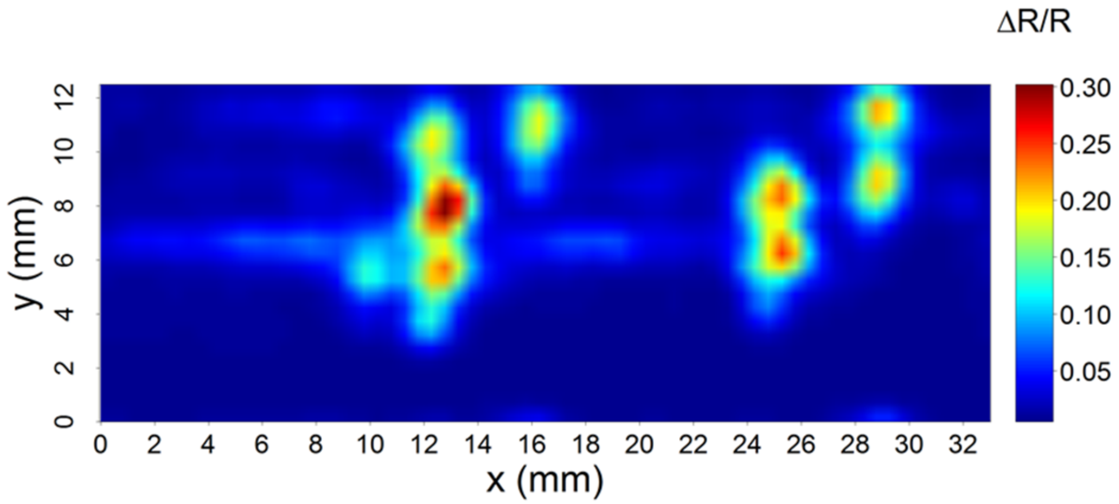

Supplementary Figure S19. Normalized change in resistance from the baseline before each touch after convolution with 2D Gaussian filter (σ = 1 pixel).

Images from Strips on Same Membrane

Supplementary Figure S20 shows images from all three strips on one sensing skin; the center strip produced Figure 4b in the main paper. While the outer strips malfunctioned slightly at the end of the scan, the same basic response is visible in all three. The relative lateral offset of the images corresponds to the distance between the sensing strips.

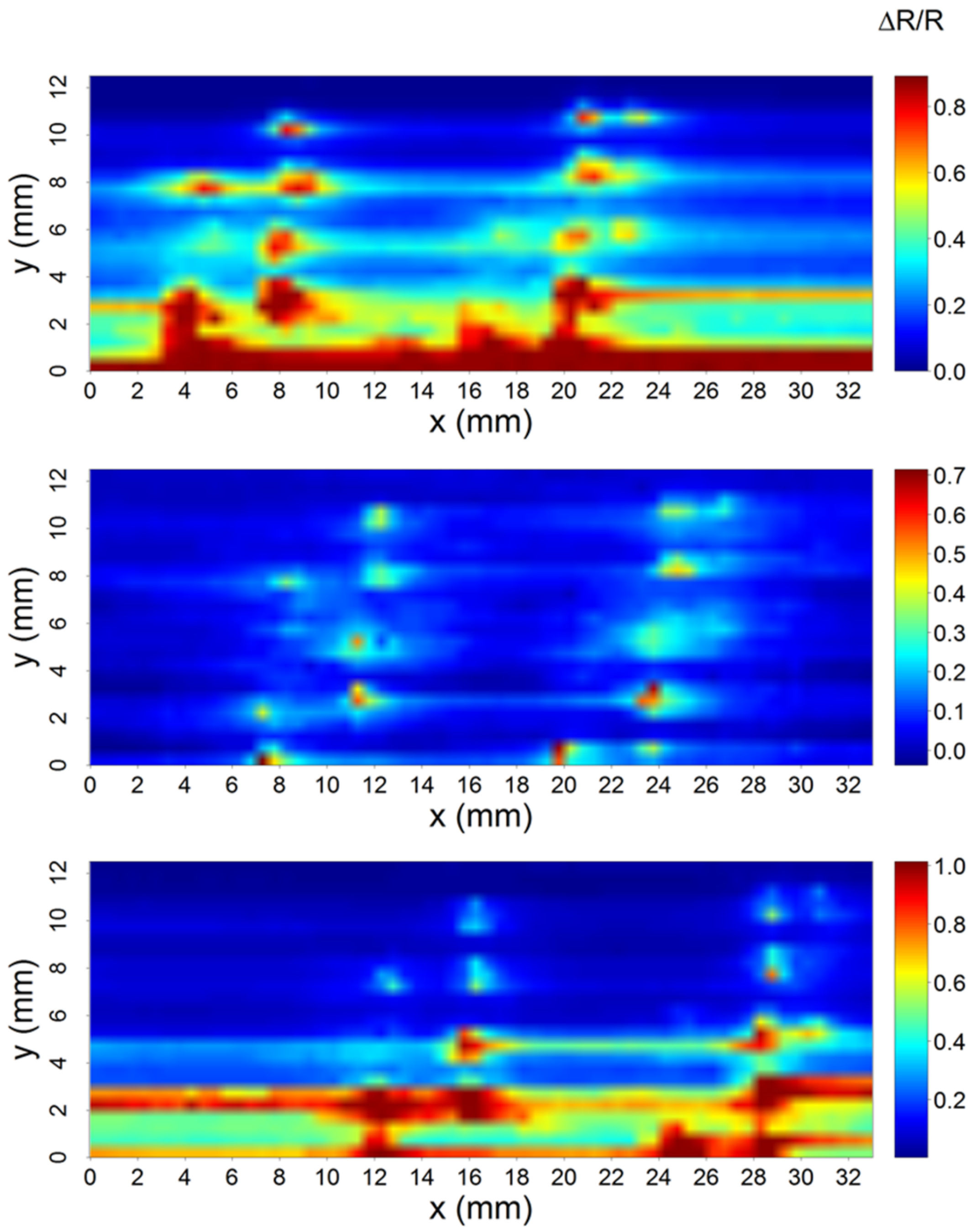

Supplementary Figure S20. Results from three strips on same membrane during same scan.

Images from Finger with an Insert

Another type of finger was fabricated after the finger with the 5 mm wide groove and before the 3D printed finger. In this finger, the foam was replaced with an insert that reduced the width of the groove to 1.6 mm, comparable to the width of one braille bump (Supplementary Figure S21). The rigid insert was laser-cut from Delrin, and then ground on a bench grinder to its final profile. The groove in the insert was left empty.

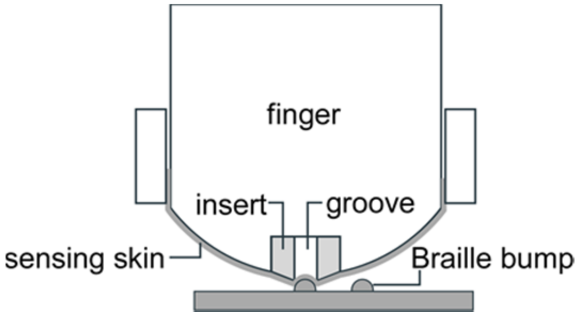

Supplementary Figure S21. Schematic illustrating the finger with an insert.

Images were produced with the second finger from four sensing skins using the same discrete scan procedure. The best result is shown in Supplementary Figure S22b. The peak positions again perfectly corresponded to the braille pattern. The peaks had a larger diameter, close to the diameter of the braille dots. As shown in the main text, this indicates that the depth of contact was greater in this scan. There was significant streaking, with the signal not returning to the baseline for quite some time after the membrane was deflected by a dot, also consistent with the greater indentation. It should be emphasized that using materials with lower viscoelasticity would reduce or eliminate the streaking, but regardless, the pattern could still be readily identified.

There was again a ghost image, albeit fainter than for the first finger, offset in the *y*-direction by 2.0 mm. The hypothesis for the ghost features in this image is illustrated in the corresponding schematic, Supplementary Figure S22a. A strong signal was generated from the good localization produced with the narrow groove. However, the machined insert had been hand-made and inserted, and it did not fit perfectly flush with the surface. Therefore, there were sections over which the membrane was not fully supported, but was suspended with a gap between the insert and the rest of the finger. When the dot deflected the membrane at this location, a faint ghost image resulted.

To test this supposition, the depth of contact was reduced by decreasing the distance travelled by the *z*-actuator. This was expected to eliminate the contact of the braille dot with the membrane suspended over the outer edge of the insert. The resulting image is shown in Supplementary Figure S22d. The ghosting was considerably reduced, without significantly diminishing the primary signal, and the dot diameter decreased slightly. The resolution between dots also improved by reducing the horizontal streaking: smaller deflections result in less viscoelastic deformation. The two closely spaced dots on the lower left slightly blended together, indicating their separation was approaching the limit of the resolution. The resolution could be improved by making the groove narrower, although that would also reduce the signal amplitude. For this application, in which the size of the dots is known, the presence of two features is clear.

These results corroborate the conclusion that if the underlying geometry allows for strains in more than one place, a ghosted image will be generated. A smooth finger is thus required that perfectly supports the membrane so that no artifacts are introduced.

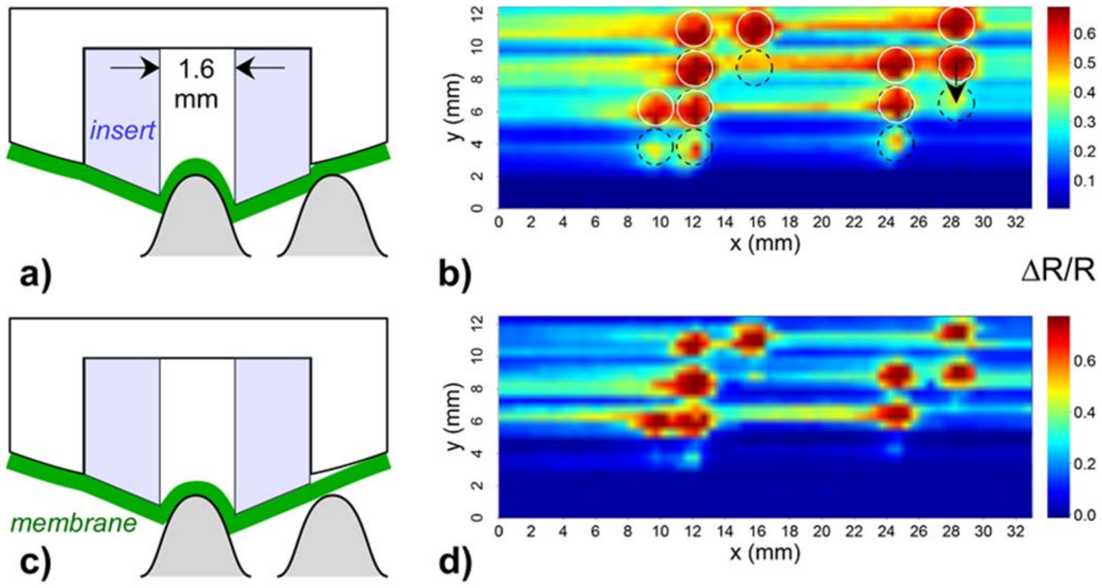

Supplementary Figure S22. Schematic representations of a finger having a groove with an insert interaction with a braille dot at a) one depth of contact and c) less depth of contact. b) The image corresponding to (a), showing ghosting. d) The image corresponding to (c) without ghosting.

Discussion of the Materials

Robustness is important for tactile sensors. The particular latex membranes used here are a shortcoming of the current prototypes. Both types of membrane use in this work degraded rapidly (within a week) upon clamping, becoming sticky and soft. Latex is also known to wear away under extended contact [1]. In future work, the latex sheet should be replaced with a more robust membrane material.

The membranes used in this work experienced viscoelastic deformation, leading to streaky images. Appropriate corrections to the baseline based on the scanning history may mitigate this (Supplementary Figure S17), or the viscoelastic effect could be handled with software [2], but a more elastic material may be preferable. We had previously characterized the sensing membrane [3], but in future work, researchers will no doubt use other types of sensors, other elastomeric materials, and other methods of fabricating the cavity. Therefore, instead of focusing on the sensor performance, we focused on understanding how the finger geometry and interaction with the surface affected the images, and we provided general guidelines for design and materials.

Although rubber fingertips provide advantages in gripping tasks, stickiness is not ideal for scanning, since it leads to membrane strain, and thereby interfering signals. The combination of stretchability and the ability to slide has been achieved in silicone rubber skin by creating a matte surface finish [4]. Coatings or an inherently less tacky membrane material could also be implemented.

The membranes were held taut over the finger surface with two metal clamping plates (Figure 2d). At times the handling and clamping process damaged the sensors or the connections, rendering some of the sensing strips inoperable. The clamping method should be further investigated.

Possible Future Directions

The cavity shape under the membrane could be specialized to target shapes of a given size, such as a hexagonal bolt head, or an interior occlusion could be added for detecting rings. This shape recognition would not require an array. There is also a practical consideration concerning wiring. A strip sensor permits electrical connections to be made at the back or side of the finger.

In the variation of the device illustrated in Figure 8c, a round bump would likely be the most typical. However, the spatial matching could in principle be made different along the x and y axes by using an oval-shaped bump. Such a sensor would additionally detect steps, since it would compress one side of the membrane, which the current configuration cannot do. In principle one could combine bumps and divots on the same fingertip.

Arduino Codes

Running a Discrete Scan

```
/* This is a test routine that will scan a braille plate with a 0.5mm grid of touches.
*/

#include <Wire.h>
#include <Adafruit_MotorShield.h>
#include "utility/Adafruit_PWMServoDriver.h"
#include <Servo.h>
#include <PSTestRig.h>

PSTestRig testRig = PSTestRig();
boolean isStarted, toggle;
unsigned long startTime;
int i,j,x,y;

void setup() {
  //Initialize test rig, set constants for scan
  x = 25;
  y = 66;
  testRig.init();
  isStarted = false;
  toggle = false;
  testRig.zHome();
}

void loop() {
  //Start when button is pressed
  if(testRig.startSwitchIsPressed() && !isStarted) {
    isStarted = true;
    for (i=1;i<=x;i++){
      //Loop over x coordinates (actually y coordinates in plot, oops)
      for (j=1;j<=y;j++){
        //Loop over y coordinates
        Serial.println("TOUCHING POINT: " + (String)x + ", " + (String)y);
        startTime = millis();
        testRig.zMoveAbsolute(.5); //Touch braille plate
        delay(500);
        testRig.zHome(); //Retract
        testRig.yMoveIncremental(.5); //Move to next touch point
        while((millis() - startTime) <= 14000) {
          //Wait to keep timing consistent for data processing
          delay(1);
        }
      }
      startTime = millis();
      testRig.yMoveAbsolute(0); //Return to start of line
      testRig.xMoveIncremental(.5); //Move to next line
      testRig.zHome(); //Be extra sure that it's retracted
```

```
      while((millis() - startTime) <= 100000) {
        //Timing again
        delay(1);
      }
    }
    //Reset to initial position at end of scan
    testRig.xMoveAbsolute(0);
    testRig.yMoveAbsolute(0);
    testRig.zHome();
    Serial.println("BRAILLE SCAN FINISHED, RESETTING TO INITIAL POSITION");
  }

  if (isStarted && !testRig.startSwitchIsPressed()) {
    isStarted = false;
  }

  //Serial.println(testRig.startSwitchIsPressed());
}
```

Running a Continuous Scan

```
#include <Wire.h>
#include <Adafruit_MotorShield.h>
#include "utility/Adafruit_PWMServoDriver.h"
#include <Servo.h>
#include <PSTestRig.h>

PSTestRig testRig = PSTestRig();
int i, x, y;
unsigned long startTime, sweepTime, stepTime;
boolean isStarted;

void setup() {
  //Initialize test rig, set constants for scan
  testRig.init();
  x = 33;
  y = 12.5;
  isStarted = false;
  sweepTime = 50000;
  stepTime = 110000;
  testRig.zHome();
}

void loop() {
  //Start scan when button pressed
  if(testRig.startSwitchIsPressed() && !isStarted) {
    isStarted = true;
    for(i=0;i<=(y*2);i++){
      startTime = millis();
      testRig.xMoveIncremental(x); //Scan forward
```

```
      while((millis() - startTime) <= sweepTime) {
        //Make sure timing is consistent for data processing
        delay(1);
      }
      testRig.xMoveAbsolute(0); //Scan backwards
      while((millis() - startTime) <= (sweepTime * 2)) {
        //Timing again
        delay(1);
      }
      testRig.yMoveIncremental(.5); //Move to next line
      while((millis() - startTime) <= stepTime) {
        //Timing again
        delay(1);
      }
    }
    //Reset to initial position
    testRig.xMoveAbsolute(0);
    testRig.yMoveAbsolute(0);
    isStarted = false;
  }

  testRig.serialExecute();
}

void serialEvent() {
  testRig.serialParse();
}
```

R Codes

Discrete Scan

```
library(EBImage)
library(plot3D)

#Read in raw voltage data from file.  File contains time-series data for each sensing strip in columns 2-4, plus time in column 1
dat<-read.csv('datafilename.txt')

#Set period length (in samples, current sampling rate 500Hz), delay between touches, and size of scanned array
period<-6999
delay<-49990
x<-66
y<-25

#Remove beginning of data stream to avoid wonkiness from when SignalExpress starts recording
truncdat<-dat[-1:-99,]

#Create empty arrays for data.
griddat<-array(dim=c(x,y,period,3)) #array containing time-series data for each grid position
gridpeaks<-array(dim=c(x,y,3)) #array containing peaks of time-series data for each grid position

#partition truncdat into separate time-series data streams for each grid location, and place in griddat
for (i in 1:x)
  for (j in 1:y)
    griddat[i,(y-j+1),,]<-as.matrix(truncdat[(((i - 1) + (j - 1)*x)*period + 1001 + (j-1)*delay):((i + (j - 1)*x)*period + (j-1)*delay + 1000),2:4])

#Convert griddat from voltage to resistance (KOhm)
griddat[,,,]<-apply(griddat, 4, function(x) (30*x)/(5-x))

#subtract off pre-scan baseline and normalize
for (i in 1:3)
  griddat[,,,i]<-(griddat[,,,i] - griddat[1,25,100,i])/griddat[1,1,100,i]

#Take peaks for each grid location in griddat
gridpeaks<-apply(griddat, c(1,2,4), max)

#Drop data below a certain threshold
#gridpeaks[gridpeaks < ((max(gridpeaks[,,1]) - min(gridpeaks[,,1]))*(3/4) + min(gridpeaks[,,1]))]<-((max(gridpeaks[,,1]) - min(gridpeaks[,,1]))*(3/4) + min(gridpeaks[,,1]))

#rotate gridpeaks 180 degrees
for (i in 1:3) {
  temp<-t(gridpeaks[x:1,,i])
```

```
  gridpeaks[,,i]<-t(temp[y:1,])
}

#Plot gridpeaks
png("plotname.png", 2800, 1332)
par(mar=c(20,20,20,20), mgp=c(10,4,0))
image2D(gridpeaks[,,3], xlab='x (mm)', ylab='y (mm)', clab=expression(paste(Delta,'R/R')),
axes=FALSE, cex.lab=7, asp=(y/x), rasterImage=TRUE, colkey=list(cex.axis = 5, cex.clab=6))
axis(1, at=seq(0,1, by=4/x), labels=seq(0,x/2,by=2), cex.axis=5)
axis(2, at=seq(0,1, by=4/y), labels=seq(0,y/2,by=2), cex.axis=5)
dev.off()

#reset griddat for second calculation
#partition truncdat into separate time-series data streams for each grid location, and place in
griddat
for (i in 1:x)
  for (j in 1:y)
    griddat[i,(y-j+1),,]<-as.matrix(truncdat[(((i - 1) + (j - 1)*x)*period + 1001 + (j-1)*delay):((i + (j -
1)*x)*period + (j-1)*delay + 1000),2:4])

#Convert griddat from voltage to resistance (KOhm)
griddat[,,,]<-apply(griddat, 4, function(x) (30*x)/(5-x))

#Take "baseline" for each grid location in griddat consisting of average of first 400 samples
base<-apply(griddat[,,1:400,], c(1,2,4), mean)

#Subtract baseline from each grid location in griddat, then normalize by the baseline value, to
compute DR/R for each touch
for (i in 1:x)
  for (j in 1:y)
    for (k in 1:period)
      griddat[i,j,k,]<-((griddat[i,j,k,]-base[i,j,])/base[i,j,])

#Take peaks for each grid location in griddat
gridpeaks<-apply(griddat, c(1,2,4), max)

#(Optional) Truncate DR/R data to improve plot quality if range is too large
#gridpeaks[gridpeaks > .2] <- .2

#Plot gridpeaks
png("plotname.png", 2800, 1332)
par(mar=c(20,20,20,20), mgp=c(10,4,0))
image2D(gridpeaks[,,2], xlab='x (mm)', ylab='y (mm)', clab=expression(paste(Delta,'R/R')),
axes=FALSE, cex.lab=7, asp=(y/x), rasterImage=TRUE, colkey=list(cex.axis = 5, cex.clab=6))
axis(1, at=seq(0,1, by=4/x), labels=seq(0,x/2,by=2), cex.axis=5)
axis(2, at=seq(0,1, by=4/y), labels=seq(0,y/2,by=2), cex.axis=5)
dev.off()


#Create and convolve with filter
ker<-makeBrush(size=15,shape='Gaussian', sigma=1)
```

```
ker<-ker/sum(ker)
img<-filter2(gridpeaks,ker)

#Plot gridpeaks
png("plotname.png", 2800, 1332)
par(mar=c(20,20,20,20), mgp=c(10,4,0))
image2D(img[,,2], xlab='x (mm)', ylab='y (mm)', clab=expression(paste(Delta,'R/R')),
axes=FALSE, cex.lab=7, asp=(y/x), rasterImage=TRUE, colkey=list(cex.axis = 5, cex.clab=6))
axis(1, at=seq(0,1, by=4/x), labels=seq(0,x/2,by=2), cex.axis=5)
axis(2, at=seq(0,1, by=4/y), labels=seq(0,y/2,by=2), cex.axis=5)
dev.off()
```

Continuous Scan

```
library(plot3D)
library(EBImage)

#Read voltage data from file
dat<-read.csv("datafilename.txt")

#Set constants to match arduino code (time values fudged slightly to compensate for clock
error)
sweepTime<-24994
stepTime<-54986
x<-66
y<-25

#Empty image arrays
fwdimg<-array(dim=c(sweepTime,y-1))
bckimg<-array(dim=c(sweepTime,y-1))


#Populate image arrays with data
for(i in 1:(y-1)) {
  fwdimg[,i]<-dat[(i*stepTime + 1):(i*stepTime + sweepTime),3]
}

for(i in 1:(y-1)) {
  bckimg[,i]<-dat[(i*stepTime + sweepTime + 1):(i*stepTime + 2*sweepTime),3]
}

#Convert from V to R
fwdimg<-30*fwdimg/(5-fwdimg)
bckimg<-30*bckimg/(5-bckimg)

#Convert from R to DR/R
fwdimg<-(fwdimg-fwdimg[100,1])/fwdimg[100,1]
bckimg<-(bckimg-bckimg[100,1])/bckimg[100,1]

#Plot
```

```
png("forwardplotname.png", 2800, 1305)
par(mar=c(20,20,20,20), mgp=c(10,4,0))
image2D(fwdimg, xlab='x (mm)', ylab='y (mm)', clab=expression(paste(Delta,'R/R')),
axes=FALSE, cex.lab=7, asp=((y-1)/x), rasterImage=TRUE, colkey=list(cex.axis = 5,
cex.clab=6))
axis(1, at=seq(0,1, by=4/x), labels=seq(0,x/2,by=2), cex.axis=5)
axis(2, at=seq(0,1, by=4/(y-1)), labels=seq(0,(y-1)/2,by=2), cex.axis=5)
dev.off()

png("reverseplotname.png", 2800, 1305)
par(mar=c(20,20,20,20), mgp=c(10,4,0))
image2D(bckimg[sweepTime:1,], xlab='x (mm)', ylab='y (mm)',
clab=expression(paste(Delta,'R/R')), axes=FALSE, cex.lab=7, asp=((y-1)/x),
rasterImage=TRUE, colkey=list(cex.axis = 5, cex.clab=6))
axis(1, at=seq(0,1, by=4/x), labels=seq(0,x/2,by=2), cex.axis=5)
axis(2, at=seq(0,1, by=4/(y-1)), labels=seq(0,(y-1)/2,by=2), cex.axis=5)
dev.off()
```

References

1 Edwards, J., Lawry, J., Rossiter, J. & Melhuish, C. Extracting textural features from tactile sensors. *Bioinspir. Biomim.* **3**, doi: 10.1088/1748-3182/3/3/035002 (2008).

2 Yousef, H., Boukallel, M. & Althoefer, K. Tactile sensing for dexterous in-hand manipulation in robotics - A review. *Sens. Act. A* **167**, 171-187 (2011).

3 Chen, Y., Yu, M., Bruck, H. A. & Smela, E. Stretchable touch-sensing skin over padding for co-robots. *Smart Mater. Struct.* **25**, 055006, doi: 10.1088/0964-1726/25/5/055006 (2016).

4 Howe, R. D. & Cutkosky, M. R. Dynamic tactile sensing: Perception of fine surface features with stress rate sensing. *IEEE Trans. Robotic Autom.* **9**, 140-151, doi: 10.1109/70.238278 (1993).
